# A plant’s perception of growth-promoting bacteria and their metabolites

**DOI:** 10.3389/fpls.2023.1332864

**Published:** 2024-01-24

**Authors:** Renée Abou Jaoudé, Francesca Luziatelli, Anna Grazia Ficca, Maurizio Ruzzi

**Affiliations:** Department for Innovation in Biological, Agrofood and Forest Systems (DIBAF), University of Tuscia, Viterbo, Italy

**Keywords:** plant growth-promoting rhizobacteria, plant growth, plant biostimulants, plant ecophysiology, leaf gas exchange, metabolome

## Abstract

Many recent studies have highlighted the importance of plant growth-promoting (rhizo)bacteria (PGPR) in supporting plant’s development, particularly under biotic and abiotic stress. Most focus on the plant growth-promoting traits of selected strains and the latter’s effect on plant biomass, root architecture, leaf area, and specific metabolite accumulation. Regarding energy balance, plant growth is the outcome of an input (photosynthesis) and several outputs (i.e., respiration, exudation, shedding, and herbivory), frequently neglected in classical studies on PGPR-plant interaction. Here, we discuss the primary evidence underlying the modifications triggered by PGPR and their metabolites on the plant ecophysiology. We propose to detect PGPR-induced variations in the photosynthetic activity using leaf gas exchange and recommend setting up the correct timing for monitoring plant responses according to the specific objectives of the experiment. This research identifies the challenges and tries to provide future directions to scientists working on PGPR-plant interactions to exploit the potential of microorganisms’ application in improving plant value.

## Graphical Abstract

Proposed classification of PGPR according to classical and ecophysiological studies. (image created using BioRender).
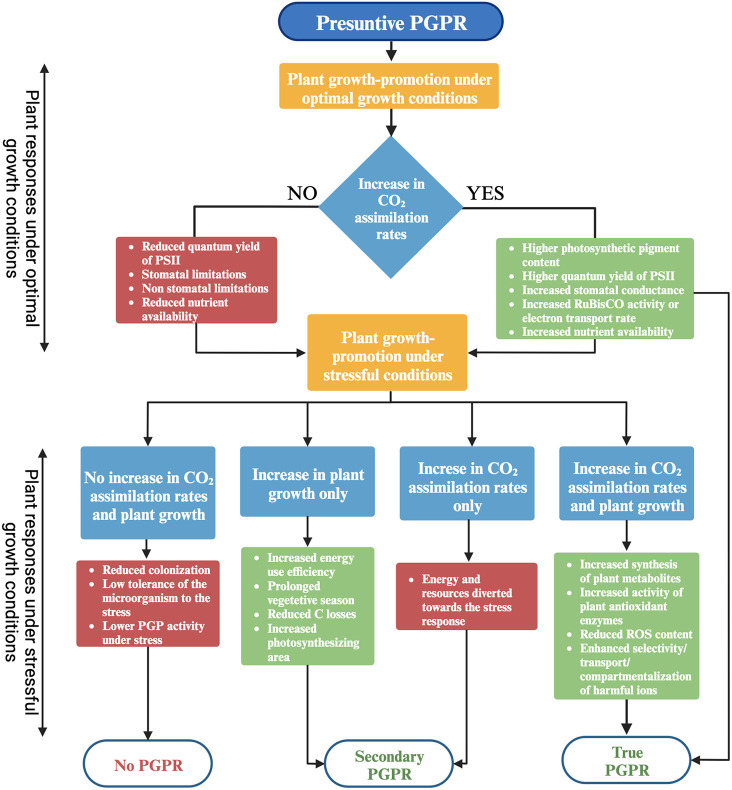

## 1 Introduction

Since 1950, the world’s population has almost tripled, reaching 8 billion people by the end of 2022, while it is expected to rise to over 10 billion by 2059 (UN, 2022). The increase in human population combined with the negative effects of climate change and pollution have meant a decline in *per capita* agricultural land available for crops and animal husbandry (FAO, 2021). Therefore, to meet the future food demand, new methods aiming at increasing productivity and supporting plant growth in marginal areas and under harsh climatic conditions while reducing chemicals need to be explored (Ruzzi and Aroca, 2015; Rahman et al., 2022).

Biostimulants are microbial or non-microbial products, generally applied to plants in small quantities, that stimulate their development by enhancing nutrient use efficiency and increasing the availability of confined nutrients in soil or rhizosphere, improve tolerance to abiotic stress, or augment plant quality traits (Kauffman et al., 2007; Du Jardin, 2015; EU, 2019). Microbial biostimulants (bacteria, archaea, fungi, viruses, and protists) are characterized by a high metabolic diversity and can provide beneficial molecules that induce positive plant physiological responses, particularly under biotic and abiotic stress (Orozco-Mosqueda et al., 2021; Gamalero et al., 2022). Their use can decrease the need of applying fertilizers, pesticides, and herbicides (Fiodor et al., 2021; Poudel et al., 2021; Pantoja-Guerra et al., 2023). Moreover, they can also contribute to a rise in carbon (C) allocation to the soil by enhancing above- and below-ground biomass and influencing C storage and sink capacity, inducing positive feedback to climate change mitigation (Lange et al., 2015; Jansson et al., 2021). Consequently, soil or plant inoculation with growth promoting microorganisms represents a sustainable bio-tool aiming at increasing plant productivity and reducing losses in various environments.

Plant growth-promoting bacteria (PGPB) represent one class of microbial biostimulants. They can inhabit the phyllosphere (leaves, stems, flowers, or fruits surfaces; phyllobacteria), colonize the plant tissues (endophytes), or live in the rhizosphere or the rhizoplane and have evolved the ability to affect plants’ physiological performances beneficially (Grover et al., 2021; Orozco-Mosqueda et al., 2021). Among phytomicrobiomes, the microbial community associated with roots is the most abundant and diverse (Backer et al., 2018). In fact, roots represent a nutrient-rich habitat for microorganisms, comprising PGPB, which can colonize the ecto- and endo- rizosphere and the rhizoplane.

In the recent years, a wide amount of research has been focused on assessing the use of these microbes in supporting plant growth (**Table 1**). PGPB and plant growth promoting rhizobacteria (PGPR) first appeared in the bibliography at the beginning of the 1980s, with more than 100 documents per year produced by the scientific community starting from 2008, particularly in 2018-2022 (Scopus, September 2023). The expressions “plant growth promoting microorganisms” and “plant growth promoting endophytes” are less used in the literature. In contrast, “plant growth promoting phyllobacteria” (referring to PGP bacteria inhabiting the phyllosphere) does not exist. However, about 100 papers on using PGPB isolated from or applied to plant leaves were published, 20% of which in the last year (**Table 1**). The main topics addressed by these research articles and reviews are the isolation of PGP bacteria and the description of their properties and potential role in alleviating plant abiotic and biotic stresses or being effective in bioremediation. The principal parameters measured to assess the effects of PGPR application are plant biomass or yield (Kang et al., 2014; Paungfoo-Lonhienne et al., 2019), while other studies monitor changes in plant morphological and functional traits (Larcher et al., 2003; Ferreira Rêgo et al., 2014). However, growth, which is the increment in biomass gain in plants and the consequent increase in agronomic yield, is the result of highly complicated processes that are directly related to the amount of carbon dioxide (CO_2_) fixed during the photosynthetic process, the way plants allocate this C and the losses through respiration, exudation, volatilization, and other negative balance sheet items, such as herbivory or shedding herbivory (Chapin et al., 2006; Collalti et al., 2020; Hilty et al., 2021). Therefore, the analysis of the result of this balance (biomass) does not give any information about the effects that PGPR can induce on the single terms of the equation, reducing the possibilities of selection and improvement of PGP bacteria with specific promoting traits.

**Table 1:** Keywords used for search within “Article title, Abstract, Keywords” on Scopus, number of documents produced by the research, and percentage of the specific research compared to the total number of documents found respectively for “Plant growth promoting rhizobacteria” and “Plant growth promoting bacteria”.

Keywords for search within “Article title, Abstract, Keywords”			No. of documents	%
Plant growth promoting rhizobacteria			5739	
Plant growth promoting bacteria			3846	
Plant growth promoting microorganisms			410	
Plant growth promoting phyllobacteria			0	
Plant growth promoting endophytes			102	
Plant growth promoting rhizobacteria	Phyllosphere		20	
Plant growth promoting bacteria	Phyllosphere		56	
Plant growth promoting rhizobacteria	Photosynthesis		170	2.96
Plant growth promoting bacteria	Photosynthesis		244	6.34
Plant growth promoting rhizobacteria	Assimilation rate		12	0.21
Plant growth promoting bacteria	Assimilation rate		9	0.23
Plant growth promoting rhizobacteria	Carbon dioxide assimilation		0	0.00
Plant growth promoting bacteria	Carbon dioxide assimilation		1	0.03
Plant growth promoting rhizobacteria	CO_2_ assimilation		9	0.16
Plant growth promoting bacteria	CO_2_ assimilation		4	0.10
Plant growth promoting rhizobacteria	Photosynthetic rate		72	1.25
Plant growth promoting bacteria	Photosynthetic rate		42	1.09
Plant growth promoting rhizobacteria	Gas exchange		52	0.91
Plant growth promoting bacteria	Gas exchange		37	0.96
Plant growth promoting rhizobacteria	Carbon assimilation		4	0.07
Plant growth promoting bacteria	Carbon assimilation		7	0.18
Plant growth promoting rhizobacteria	Photosynthetic rate	Stomatal conductance	29	0.51
Plant growth promoting bacteria	Photosynthetic rate	Stomatal conductance	14	0.36

Please note that the exact expressions, and not the single words, were used in the research process. The research data is current as of September 2023.

To date, less than 10% of the available research papers have focused on measuring photosynthesis (CO_2_ assimilation rate) and photosystem functionality, and many research articles have concentrated their attention on just one aspect of the photosynthetic activity (**Tables 1**, **2**). However, photosynthesis is a complicated process, and a partial analysis of the main parameters involved in CO_2_ assimilation can lead to insufficient, misleading, or non-univocal interpretations.This review critically analyzes the literature regarding the effect of PGPR and PGPB on plant growth and sensing, under stressful and non-stressful conditions. Herein, we discuss the importance of using leaf gas exchange measurement to assess the modifications triggered by PGP bacteria and their metabolites on different aspects of the photosynthetic process. Finally, we highlight the gaps in the literature and identify the challenges to provide future directions to scientists working on bacteria-plant interactions to exploit the potential of PGP microorganisms’ application in improving plant value. Our objective is to point out the controversies that can derive from the consideration of an insufficient number of photosynthetic variables, the main evidence regarding the effect of PGP bacteria on leaf gas exchange under optimal plant growth conditions and in stressed plants, the importance of choosing the duration of the experiment, the type of PGPB and the concentration at which it is applied.

**Table 2:** Effects of PGPR inoculation on photosynthetic parameters measured in plants grown under non-stress conditions, compared to non-inoculated plants (↑: increase; ↓: decrease; = non-significant variations).

Plant species	Plant Growth-Promoting Strain	Parameter	PGPB vs. Control	Reference
*Arabidopsis thaliana*	*Staphylococcus* sp. I26^a,k,m^ *Bacillus* sp. L81^m^ *Curtobacterium* sp. M84^k^ *Arthrobacter oxydans* BB1^h,m^	F_v_/F_m_	I26=	Barriuso et al., 2008
			L81↑	
			M84=	
			BB1=	
*Arabidopsis thaliana*	*Bacillus subtilis* GB03^g,n^	F_v_/F_m_	↑	Zhang et al., 2008
		Ф_PSII_	↑	
		NPQ	↓	
		Chlorophyll a/b	=	
		Total chlorophyll	↑	
*Arabidopsis thaliana*	*Bacillus subtilis* GB03^g,n^	Total chlorophyll	↑	Xie et al., 2009
*Arabidopsis thaliana*	*Phyllobacterium brassicacearum* STM196^r^	CO_2_ assimilation rate	↓	Bresson et al., 2013
		Night transpiration rates	↓	
		Transpiration rates	↓	
		Water use efficiency	=	
*Glycine max*	*Pseudomonas fluorescens* N21.4^d,m^ *Stenotrophomonas maltophilia* N5.18^d,m^ *Chryseobacterium balustinum* Aur9^h^ *Curtobacterium* sp. M84^k^	Ф_PSII_	N21.4↓	Algar et al., 2014
			N51.8↓	
			Aur9↓	
			M84=	
*Glycine max*	*Pseudomonas* sp. AK-1^a,f,h,k,m^ *Bacillus* sp. SJ-5^a,f,h,k,m^	Total chlorophyll	AK-1=	Kumari et al., 2015
			SJ-5=	
*Trigonella foenum-graecum*	Consortium of *Azotobacter chroococcum* *Enterobacter asburiae* *Lactococcus lactis*	CO_2_ assimilation rate	↑	Bisht et al., 2022
		Stomatal conductance	↑	
		Transpiration rates	↑	
		Intercellular CO_2_ conc.	↑	
		Carotenoids	↑	
		Chlorophyll a	↑	
		Chlorophyll b	=	
		Total chlorophyll	↑	
*Cicer arietinum*	*Mesorhizobium ciceri* (MC)^a,f,j,k,m^ *Serratia marcescens* SF3^a,f,k,m^ *Serratia* sp. ST9^a,f,k,m^	Total chlorophyll	MC=	Shahzad et al., 2014
			SF3=	
			ST9=	
			MC + SF3↑	
			MC + ST9↑	
		CO_2_ assimilation rate	MC=	
			SF3=	
			ST9=	
			MC + SF3↑	
			MC + ST9↑	
		Transpiration rates	MC=	
			SF3↑	
			ST9↑	
			MC + SF3↑	
			MC + ST9↑	
*Phaseolus coccineus*	*Bacillus pumilus* S4^k,m^ *Bacillus mycoides* S7^h^	CO_2_ assimilation rate	S4=	Stefan et al., 2013
			S7 (day 28-42)↑	
			S4+S7 (day 28-42)↑	
		Transpiration rates	S4=	
			S7=	
			S4+S7 (day 28)↑	
		Water use efficiency	S4=	
			S7 (day 28-96)↑	
			S4+S7 (day 28-42)↑	
		Total chlorophyll	S4 (day 28)↑	
			S7=	
			S4 + S7=	
*Panicum virgatum*	*Burkholderia phytofirmans* PsJN^a,h^	CO_2_ assimilation rate	↑	Wang et al., 2015
		Stomatal conductance	↑	
		Water use efficiency	↑	
		Ci/Ca	↓	
*Triticum aestivum*	Consortium of *Bacillus* sp.^e,h,p,q^ *Azospirillum lipoferum^e,h,p,q^* *Azospirillum brasilense^e,h,p,q^*	CO_2_ assimilation rate	↑	Akhtar et al., 2021
		Transpiration rates	↑	
		Stomatal conductance	↑	
		Chlorophyll a	↑	
		Chlorophyll b	↑	
		Carotenoids	↑	
*Zea mays*	*Pseudomonas fluorescens* Aur6^h,m^	F_0_	=	Grijalbo et al., 2013
		F_v_/F_m_	=	
		Hill reaction	↑	
		Chlorophyll a/b	↑	
		Total Chlorophyll	↑	
*Zea mays* cv Marzuka *Zea mays* cv Kaleo	*Burkholderia phytofirmans* PsJN^a,h^ *Enterobacter* sp. FD17^a,b,c,k^	CO_2_ assimilation rate	PsJN both cv↑	Naveed et al., 2014
			FD17 both cv↑	
		Stomatal conductance	PsJN both cv↑	
			FD17 both cv=	
		Transpiration rates	PsJN both cv↑	
			FD17 both cv↑	
		Vapour pressure deficit	PsJN both cv=	
			FD17 both cv=	
		Relative water content	PsJN both cv↑	
			FD17 both cv↑	
		F_v_/F_m_	PsJN both cv↑	
			FD17 both cv=	
		Total chlorophyll	PsJN both cv↑	
			FD17 both cv↑	
*Zea mays*	*Bacillus megaterium* ^s^	Stomatal conductance	=	Romero-Munar et al., 2023
		Ф_PSII_	=	
		CO_2_ assimilation rate	=	
		Water use efficiency	=	
		Stomatal conductance	=	
		Ф_PSII_	=	
		CO_2_ assimilation rate	=	
		Water use efficiency	=	
*Sambucus williamsii*	*Acinetobacter calcoaceticus* X128^e,h,q^	CO_2_ assimilation rate	↑	Liu et al., 2019b
		Stomatal conductance	↑	
		Intercellular CO_2_ conc.	=	
*Trema micrantha* *Cariniana estrellensis*	*Azospirillum brasilense* Ab-V5^j^ *Bacillus* sp. (BA)^h^ *Azomonas* sp. (AM) *Azorhizophillus* sp. (AR)	Water potential	Ab-V5 in *T.m.*=	Nunes Tiepo et al., 2018
			Ab-V5 in *C.e.*=	
			BA in *T.m.*=	
			BA in *C.e.*=	
			AM in *T.m.*=	
			AM in *C.e.*=	
			AR in *T.m.*↓	
			AR in *C.e.*=	
		CO_2_ assimilation rate	Ab-V5 in *T.m.*=	
			Ab-V5 in *C.e.*=	
			BA in *T.m.*=	
			BA in *C.e.*=	
			AM in *T.m.*=	
			AM in *C.e.*=	
			AR in *T.m.*=	
			AR in *C.e.*↓	
		Stomatal conductance	Ab-V5 in *T.m.*=	
			Ab-V5 in *C.e.*=	
			BA in *T.m.*=	
			BA in *C.e.*=	
			AM in *T.m.*=	
			AM in *C.e.*=	
			AR in *T.m.*=	
			AR in *C.e.*↓	
		Intercellular CO_2_ concentration	Ab-V5 in *T.m.*=	
			Ab-V5 in *C.e.*=	
			BA in *T.m.*=	
			BA in *C.e.*=	
			AM in *T.m.*=	
			AM in *C.e.*=	
			AR in *T.m.*=	
			AR in *C.e.*=	
		Carboxylation efficiency	Ab-V5 in *T.m.*=	
			Ab-V5 in *C.e.*=	
			BA in *T.m.*=	
			BA in *C.e.*=	
			AM in *T.m.*=	
			AM in *C.e.*=	
			AR in *T.m.*=	
			AR in *C.e.*=	
*Vitis vinifera*	*Burkholderia phytofirmans* PsJN^a,h^	CO_2_ assimilation rate	↑	Ait Barka et al., 2006
*Vitis vinifera*	*Burkholderia phytofirmans* PsJN^a,h^	CO_2_ assimilation rate	↓	Fernandez et al., 2012
		Stomatal conductance	=	
		Intercellular CO_2_ conc.	=	
		Ф_PSII_	=	
		Total chlorophyll	↓	
		Carotenoids	↓	
*Salicornia ramosissima*	Consortium of *Thalassospira australica* SRT8^a^ *Pseudarthrobacter oxydans* SRT15^c,h,j^ *Vibrio neocaledonicus* SRT1^c,h,j,k,m^	CO_2_ assimilation rate	=	Mesa-Marín et al., 2020
		Stomatal conductance	=	
		Intercellular CO_2_ conc.	=	
		Water use efficiency	=	
		F_m_	↓	
		F_v_/F_m_	↓	
		Absorbed energy flux	=	
		Trapped energy flux	=	
		ET energy flux*	=	
		Dissipated energy flux	↑	

*Electron transport energy flux.

a ACC deaminase.

b Biocontrol.

c Biofilm formation.

d Chitinases production.

e Cytokinins production.

f Exopolysaccharide production.

g Glucose/ABA sensing.

h IAA production.

i Induction of systemic resistance.

j N fixation.

k Phosphate solubilization.

l Phytase production.

m Siderophore production.

n VOCs production.

°Cd tolerance.

p ABA production.

q Osmolyte production.

r Effect on root architechture.

s Effect on root hydraulic properties.

## 2 Plant growth in a changing environment

Plant growth can be defined as the increase in size, volume, or biomass resulting from two main cellular mechanisms: proliferation and cell expansion (Lambers and Oliveira, 2019). Cell proliferation determines an increase in the cell number. In contrast, cell expansion, occurring after proliferation, determines variations in the cell size/volume: elongation, extension (i.e., the development in one direction), and enlargement (i.e., the development in three dimensions) (Barrada et al., 2015; Harris et al., 2016; Oh et al., 2020).

Proliferation in plants is a lifetime continuous process driven by meristems which are a source of cells that differentiate and form new tissues (histogenesis) and organs (organogenesis): root and shoot primary meristems, and secondary meristems (axillary and intercalary meristems, meristemoids and lateral vascular and cork cambium) (Ramirez-Parra and Gutierrez, 2005; Sisodia and Bhatla, 2018). Although plants can vary in cell division rate, the increase in cell number *per se* does not determine growth, which is regulated by cell expansion (Reinhardt and Kuhlemeier, 2002; Lambers and Oliveira, 2019). While the increase in the number of cells resulting from mitotic cycles is usually not preceded by changes in vacuole volume (Majda and Robert, 2018), the directional and dimensional size variation of individual cells mostly depends on vacuole expansion through the uptake of water (Forouzesh et al., 2012; Sablowski and Dornelas, 2014). This turgor-driven extension is limited by the rigidity of the cell-wall matrix that surrounds plant cells, and which needs to be loosened to allow a volumetric modification of the cell (Forouzesh et al., 2012; Majda and Robert, 2018). The process, which is well described by Wolf et al. (2012), first requires the hydration and swelling of the cell wall, which facilitates the rearrangement of cellulose microfibrils, allowing wall extension, and activates, at the same time, a cascade of responses which ultimately result in the creation of cross-linking among the cell wall components and the consequent dehydration of the cell wall, finally followed by the deposition of new cell wall material.

Besides the complicated cellular mechanisms involved in division and expansion, it is clear that while water represents a cheap mean to increase cell size, the accumulation of cellular components due to macromolecular synthesis is cost effective and, consequently, depends on nutrient and energy availability (Sablowski and Dornelas, 2014).

Plants growing in natural environments are subjected to uncontrolled and ever-changing physical and biological conditions (Kollist et al., 2019). Therefore, the plant’s development is influenced by several abiotic (mainly light, water availability, temperature, contaminants, salinity) and biotic (herbivores, symbionts, and pathogens) factors, to which these sessile organisms must adapt and cope with, to survive (Osakabe et al., 2013).

Plant responses to changing environmental growth conditions can be positive or negative, and a specific variable’s effect on growth can affect species or biomes differently. For example, an increase in temperature in ecosystems in which a hot-dry period is already limiting plant development produces a negative effect on plant growth; however, in environments in which vegetation is limited by low temperatures, a warmer condition can induce a shift in thermal tolerance or positively alter plant phenology (Peñuelas et al., 2007). Similarly, referring to biotic factors, the plant can be affected by living organisms: positively, if it alone (commensalism) or both (mutualism) benefit from the interaction; negatively, if the plant is harmed by the living organism (parasitism); the plant can even be unaffected (neutralism) (Whipps, 2001; Wu et al., 2009).

Under sub-optimal growth conditions, both cell expansion and cell division can be limited, or, on the other hand, plants can stimulate the growth of specific tissues or organs to overcome or adapt to stress. Examples of this latter case are the production of deeper roots under reduced soil water availability (Pinheiro et al., 2005; David et al., 2007), the increase in shoot length when light is deficient (Kim et al., 2011), the emission of adventitious roots under anoxic or hypoxic soil conditions (Pedersen et al., 2021), or the substitution of less adapted with drought resistant leaves (de Dato et al., 2013). Thus, cell elongation or differentiation can represent one active strategy to displace organs in the space towards more favorable environmental conditions (Baluška et al., 2001) or provide more efficient means to limit the negative effect of the stressor on plant growth. Although these strategies usually sacrifice the development of other parts of the plant, and the whole plant biomass accumulation is often negatively affected, resulting in significant decreases in crop yield (Osakabe et al., 2013; Zhang et al., 2020), these findings suggest that the determination of the total plant biomass alone does not give precise information on the effect of one stress on plant C allocations.

Although plant responses aiming at coping with or avoiding specific stress can vary depending on the ecology of the plant and its plasticity, a common dynamic that triggers plants’ reactions to stress implies stress sensing, stress signaling, and, finally, stress-induced responses. Rapid (from seconds to minutes) physiological, biochemical, metabolic, and molecular responses to stress sensing are fundamental for the plant to face the stress and prevent irreversible damage. They can modulate slower (from minutes to hours) responses to successfully acclimate to the new environmental conditions (Kollist et al., 2019). Plants can sense environmental stimuli and regulate their growth and development responses to abiotic and biotic stresses after activating specific receptors (La et al., 2023). Reasonably, most of these receptors are in the outer borders of the vegetable cell, the cell wall, and the plasma membrane, and their activation by changing environmental conditions triggers a series of downstream signals that are transmitted to target proteins and activate transcriptional factors (Osakabe et al., 2013; Zhang et al., 2018; Kollist et al., 2019). For instance, phospholipids in the plasma membrane can be affected by changing temperature, determining a variation in the fluidity of the plasma membrane that can modify its functionality and change the structure of membrane proteins (Kollist et al., 2019); hyperosmotic stress, induced, for example, by drought or salinity, can affect plasma membrane curvature by modifying turgor pressure stimulating plasma membrane mechano-sensors (Zhang et al., 2020), while the salt overly sensitive (SOS) signaling pathway, which comprises the SOS3, SOS2, and SOS1 proteins able at detecting the increase in cytoplasmic Ca^2+^ concentration, concomitant to an increase in Na^+^ influx in the cell, activates a Na^+^/H^+^ antiporter which ultimately results in the efflux of excess ions (Ji et al., 2013; Zhang et al., 2018); an increase in cell wall peroxidases has been shown under osmotic stress in response to a rise in reactive oxygen species (ROS) concentration (Šimonovičová et al., 2004; Maia et al., 2013). The first signaling event after stress sensing, common to different stresses, is the modification of intracellular Ca^2+^ concentration and the production of secondary signaling molecules and ROS (Verma et al., 2016). The high number of biochemical pathways used by plants to protect themselves from stress activate a wide array of growth regulators and stress signaling molecules (Müller and Munné-Bosch, 2006; Roychoudhury and Aftab, 2021); thus, from embryogenesis to senescence, plant development is subjected to regulation, which is primarily mediated by phytohormones (Castro and Bucio, 2013).

Phytohormones are small biomolecules responsible for controlling cell expansion and division and regulating diverse mechanisms, mainly involved in immune reactions and responses to biotic stress (Davies, 2010; Vos et al., 2013; Altaf et al., 2023). The effect of each plant hormone depends on the plant species, target organ, rate of transport to the target tissue, and phytohormone concentration (Small and Degenhardt, 2018).

Under stress conditions, some phytohormones are known to induce an inhibition of plant growth. For example, the production of ethylene, a small gaseous molecule, is enhanced in response to multiple environmental stresses and, as reviewed by Dubois et al. (2018), the increase in the concentration of this hormone is mainly correlated with a reduction of leaf growth due to its negative effect on cell division and cell expansion. Abscisic acid (ABA) is the major phytohormone regulating developmental processes (seed maturation, seed dormancy) and stress responses (stomatal closure, leaf senescence, and growth inhibition), inducing the expression of many genes involved in the adaptation to plant stress (Zhang et al., 2020; Roychoudhury and Aftab, 2021). Stomatal closure represents one of the plants’ most rapid physical reactions under stress conditions mediated by an increase in ABA (Kollist et al., 2019). ABA triggers the accumulation of ROS in the cytoplasm of guard cells and increases the cytosolic concentration of Ca^2+^ (Liu et al., 2022), reducing turgor and inducing stomatal closure. Salicylic acid, a phenolic plant hormone, also has signaling activity during drought (Aloo et al., 2023) and may have a protective function in biotic stress tolerance. Moreover, hexokinases can also participate in the plant stress response by sensing the sugars produced during the photosynthetic process (Zhang et al., 2008). If sucrose production is not balanced by adequate phloem transport, the surplus sucrose is translocated to the stomata through the transpiration stream, activating stomatal closure via hexokinases and inhibiting photosynthesis while inducing storage processes (Granot et al., 2014).

Other hormones can play an important role in plant growth. Auxins stimulate cell division in the cambium (Davies, 2010; Rademacher, 2015; Gallei et al., 2020) and can control cell elongation by increasing osmolyte concentration and water permeability in cells while decreasing wall pressure (Keswani et al., 2020). Auxins are responsible for the apical dominance, the delay of leaf abscission, the development of flower organs and fruits, the emission of lateral roots and adventitious roots (Keswani et al., 2020; Altaf et al., 2023), although they are known to exert an inhibitory effect on primary root growth (Qin and Huang, 2018). Cytokinins regulate the synthesis of proteins involved in mitosis (Small and Degenhardt, 2018) and thus stimulate cell division in meristematic tissues (Rademacher, 2015). Furthermore, crosstalk interactions between hormones have been revealed to control cell division: auxins can cooperate with ethylene to control primary root and root hair growth and antagonistically interact with it in lateral root formation (Qin and Huang, 2018); cytokinins seem to work in pairs with auxins in the regulation of cell division, through a positive association in the growth of calli (Saini et al., 2013), or antagonistically to promote lateral plant growth rather than apex buds (George et al., 2008) and are therefore involved in the determination of apical dominance (Castro and Bucio, 2013). Regarding cell expansion, the initiation of wall slackening, its remodeling, and the following synthesis and deposition of new cell-wall components that will have to proceed contemporarily to cell expansion seem to be strictly and primarily controlled by transcriptional and non-transcriptional auxin signaling pathways (George et al., 2008; Barrada et al., 2015; Majda and Robert, 2018; Gallei et al., 2020), and secondarily by brassinosteroids (Caesar et al., 2011; Oh et al., 2020; Wang et al., 2020), cytokinins (Brault and Maldiney, 1999), and jasmonic acid (Takahashi et al., 1994). ABA has also been reported to stimulate cell expansion (Humplík et al., 2017).

Independently of plant species, an adaptive response to stress pushes the plant to allocate resources to organizing stress signaling networks or in repairing damages caused by stress (Osakabe et al., 2013): this reduces C consumption for growth (Zhang et al., 2020). One example is plant response to salinity. Salinity can lead to cellular dehydration, which causes osmotic and oxidative stress and, in turn, negatively affect cellular structures and metabolism (Bartels and Sunkar, 2005). It is well known that in saline conditions, plants accumulate solutes in their cells to reach a higher concentration compared to the external solution, to maintain a positive turgor pressure and avoid desiccation (Flowers et al., 2015). Differently from halophytes, that mainly accumulate Na^+^ and Cl^-^ to increase turgor pressure, glycophytes synthetize organic solutes which, however, have a greater energy cost compared to vacuolar compartmentalization of sodium and, even more, chloride ions (Rea and Poole, 2003). Thus, the coupled effect of the reduction in assimilation rate due to stomatal limitations, and the high cost of production of secondary metabolites usually results in a lessening of the amount of photosynthates allocated for growth (Colmer et al., 1996).

Thus, either if they are growing in optimal or stress conditions, plants must constantly evaluate the environmental and endogenous signals that impact cell division and or differentiation programs to decide where to redirect their resources (Castro and Bucio, 2013; Dubois et al., 2018). Any factor increasing the amount of available resources or reducing the cost of stress responses can be considered as an important tool for improving plant growth.

Net photosynthesis depends on the amount of energy received by Photosystem II, on the efficiency of the linear transport of electrons through the electron transport chain (the electron transport rate), on the concentration of CO_2_ at the carboxylative sites, determined by stomatal and mesophyll conductance, and on RuBisCO carboxylative activity during the Calvin-Benson cycle (Kalaji et al., 2016). Chlorophyll fluorescence reflects the first reactions occurring during the photosynthetic process in which light energy is absorbed and is only partially transferred to the photochemical reactions (Zou and Zhang, 2020), while the rest can be dissipated as heat or re-emitted as light (chlorophyll fluorescence) (Maxwell and Johnson, 2000). Chlorophyll fluorescence, measure through a fluorometer, represents a valuable tool for detecting the changes provoked in plants by external stimuli and it can be used to estimate photochemical (Ф_PSII_: quantum yield of Photosystem II or efficiency of Photosystem II photochemistry measured in illuminated leaves; F_v_/F_m_: the maximum quantum yield of Photosystem II, measured in dark-adapted leaves) and non-photochemical quenching (NFQ: the efficiency with which the energy is transformed into heat) parameters (Lambers and Oliveira, 2019). The estimation of the net CO_2_ assimilation rate, the dark respiration, the stomatal conductance, the transpiration rate, the intercellular CO_2_ concentration, and the electron transport and carboxylation rates can be measured or calculated by using gas exchange systems, which rely on IRGAs (infrared gas analyzers) to detect variations in CO_2_ and H_2_O concentrations between the air entering the leaf chamber and at the outlet (Bellasio et al., 2016; Lambers and Oliveira, 2019). The concomitant measurement of all these parameters allows the characterization of the plant status and the effect of any biotic and abiotic factor on plant growth, in a fast, non-destructive, and non-invasive way (Flexas et al., 2007). Therefore, they can efficiently be used to assess the effect of PGPR application on many important processes that drive plant growth.

## 3 Plant growth-promoting bacteria as biostimulants

The main positive effects of PGPR on plant performances can be categorized into four classes which are related to amelioration of the plant’s nutritional status, increased tolerance to abiotic stresses, prevention, and control of pathogen infections, and mitigation of the negative impact of contaminants (Ahluwalia et al., 2021; Ayuso-Calles et al., 2021; Bessai et al., 2021; Mishra et al., 2021).

The PGPR-driven improvement in plant nutritional status can be attained by increasing the availability of nutrients for plant uptake (Paungfoo-Lonhienne et al., 2019; Masood et al., 2020).

This effect can be achieved by adding new nutrients directly produced by the PGPRs (Fürnkranz et al., 2008; Moreau et al., 2019; Lorenzi et al., 2022). Nitrogen fixation by diazotrophs is one example of the role of PGPR in increasing the input of nutrients in the soil. Nitrogen is the main nutrient limiting plant growth in terrestrial ecosystems (Moreau et al., 2019). Some N-fixing microbes can convert N_2_ into NH_4_^+^, a reaction catalyzed by the enzyme nitrogenase, thus transforming N from a form unavailable to the plant into another that roots can absorb (Moreau et al., 2019). The fixation of N_2_ and its conversion to NH_4_^+^ is an energy-demanding process: the energy provided by the oxidation of one glucose molecule must almost all be devoted to producing NH_4_^+^, meaning that readily usable carbon sources must be available (Tate, 2020).

PGPR can also increase nutrient availability by modifying soil components from their less assimilable forms to more assimilable ones. Phosphorus (P) is an essential element for all living organisms, being required for the synthesis of many biologically fundamental molecules (Mackey and Paytan, 2009). In plants, it is the second most important macronutrient after nitrogen and is involved in energy transfer, cell proliferation, photosynthesis, development, and reproduction (Sashidhar and Podile, 2010; Billah et al., 2019). P is present in soils in the range of 400–800 mg kg^-1^ (Warren and Spiers, 2021) in both inorganic (primary and secondary phosphate (PO_4_^3-^) minerals; Mackey and Paytan, 2009; Fink et al., 2016) and organic (inositol phosphate, phospholipids, nucleic acids, phosphorylated pyridines, and nucleotides; Tate, 2020) fractions. The plant uptake of specific compounds of the soil P pool can be different. Inorganic phosphate compounds (H_2_PO_4_^-^ and HPO_4_^2-^) deriving from orthophosphoric acid (H_3_PO_4_) are released from primary minerals and organic debris during pedogenesis and mineralization or added to soil through P fertilization (Fink et al., 2016; Solangi et al., 2023). Plants preferably absorb P in the form of H_2_PO_4_, whose solubility is higher at a pH of 5-6 (Sharanappa, 2023); an increase in pH can induce the conversion of monovalent ions to divalent and trivalent forms characterized by a reduced solubility (Billah et al., 2019). Orthophosphate ions are very reactive: the anions can (1) tightly bind soil cations and precipitate or (2) be adsorbed to charged soil constituents (Oburger et al., 2011; Fink et al., 2016), becoming not readily available for plant uptake (labile soil P fraction). Therefore, the reduced P accessibility to plants often represents a limiting factor for plant growth, as P deficiency can affect the balance between the synthesis and catabolism of carbon metabolites (Solangi et al., 2023), independently of the application of a P fertilizer, which can also bring to the accumulation of non-available P, the so-called “legacy P” (Yu et al., 2022). P fixation in soils depends on pH and soil type (Oburger et al., 2011). Considering pH, in acidic soils, inorganic phosphorous is fixed by aluminum (Al)/iron (Fe) cations, while in alkaline soils, it is bound by calcium (Ca) cations (Billah et al., 2019). In soils with high soil organic matter, the turnover of living biomass and the mineralization of soil organic matter pools can help plants meet their P requirements (Murphy, 2014; Tate, 2020). This result can be achieved directly through the release of P from mineralized organic matter and indirectly by the negatively charged functional groups in organic substances (e.g., carboxyl, phenol) (Fink et al., 2016), which trigger a cascade of effects that ultimately bring to the increase in the concentration of orthophosphates. A similar functional group’s mediated mechanism has been reported by Oburger et al. (2011), where the ability of organic acids to solubilize P was considered under different experimental conditions; particularly, organic acid has been reported to increase the solubilization of orthophosphates. Upon acid dissociation, carboxylic acid anions (R-COO^-^) can (1) adsorb to the positively charged minerals (i.e. iron and aluminum oxides), increasing competition with PO_4_^3-^ (and other anions) for adsorption sites, decreasing mineral’s positive surface charge and weakening the strength of P adsorption, or (2) replace PO_4_^3-^ on metal oxides adsorption sites (ligand exchange), (3) exchange precipitated forms of P (Fe, Al and Ca complexes); the released H^+^ can (1) induce the negatively charged carboxyl groups to chelate positive divalent cations with the consequent release of phosphates (H_2_PO_4_^-^ or HPO_4_^2-^) from phosphatic compounds, (2) acidify the environment, leading to the dissolution of inorganic P minerals and (3) decrease the PO_4_^3-^ negative charges, thus lowering their adsorption affinity (Jones, 1998; Sashidhar and Podile, 2010; Fink et al., 2016; Billah et al., 2019). On the other hand, organic acids can also decrease inorganic phosphate availability; for example, adsorbed carboxylic acid anions can bind metal cations which can bind orthophosphate in a structure called a cation bridge (Fink et al., 2016), while protonation can make the negatively charged surfaces more positive, thus increasing the retention of orthophosphates by the matrix (Oburger et al., 2011).

Notably, the degree of complexation of metal ions by organic acids depends on the number of carboxyl groups which increases with the number of (-C(=O)OH) groups, the type of metal, and the pH (Jones, 1998; Parker et al., 2005). Moreover, sorption depends on soil type, and the sorption trend is phosphate > oxalate > citrate > malate > sulfate > acetate (Jones, 1998). Many plants have evolved the ability to secrete large quantities of organic acid from the root apparatus (Raghothama, 2020), and soil microorganisms (bacteria and fungi) inhabiting the rhizosphere have been reported to assist plants in the mobilization of insoluble phosphate products, increasing plant P uptake and, consequently, growth (Raymond et al., 2021; Wang et al., 2022). The microorganisms involved in solubilizing inorganic soil phosphates unavailable for plant uptake are globally known as Phosphate-Solubilizing Microorganisms (PSM) (Raymond et al., 2021). PSM can secrete low molecular weight organic acids, among which gluconic and 2-keto gluconic acids are the most efficient in solubilization (Sashidhar and Podile, 2010). These two acids are released to the outer cell surface by microorganisms, mainly by Gram-negative bacteria, in which glucose is oxidized to gluconate by the quinoprotein glucose dehydrogenase (PQQGDH, containing pyrroloquinoline quinone) and, possibly in some bacteria, more oxidized to 2-keto glucate by gluconate dehydrogenase (GDH) through the Entner-Doudoroff pathway (Fuhrer et al., 2005; Sashidhar and Podile, 2010).

Another example of increased plant nutrient availability driven by microorganisms is iron. Although Fe is abundant in soils, it is mainly in an insoluble form (oxyhydroxide) that is not readily accessible to plants (Zhang et al., 2009). Rhizospheric bacteria can secrete low molecular weight Fe chelators, called siderophores, which solubilize Fe from minerals and organic matter, improving Fe mobility and availability to plants (Kumawat et al., 2019; de Andrade et al., 2023).

Finally, an improvement of the plant’s nutritional status can be achieved by increasing root absorption efficiency as a result of the change in root growth and architecture (Mantelin et al., 2006; Apine and Jadhav, 2011; Ferreira Rêgo et al., 2014). Root architecture results from interactions between the plant organ and its physical, biological, and chemical environment. Thus, root traits are shaped by nutrient availability, water, and environmental gradients, and by the biotic interaction with other plants, with the soil fauna, and with microorganisms (Grover et al., 2021). PGPR have been reported to change the root architecture deeply by enhancing root elongation (Shahzad et al., 2014; Kumari et al., 2015; Wang et al., 2015; Bisht et al., 2022), belowground biomass (Ait Barka et al., 2006; Bresson et al., 2013; Naveed et al., 2014), lateral roots (Mantelin et al., 2006; Contesto et al., 2010; Kumari et al., 2015), improving root acquisition of nutrients and water (Grover et al., 2021). One of the most important microbial drivers inducing root trait modifications is the capability of certain PGP strains to excrete plant hormones, which stimulate plant growth (Castro and Bucio, 2013). A vast amount of literature has focused on microbial auxins, particularly indole-3 acetic acid, because, as mentioned before, these hormones are directly involved in plant growth control, and more than 50% of the characterized PGPR produce auxins (Khalid et al., 2004). Bacterial synthesis of IAA can be tryptophan-dependent or tryptophan-independent. The tryptophan-dependent IAA production follows five pathways; the main pathway is the indole pyruvic acid, but indole-3-acetamide, tryptophan side-chain oxidase, tryptamine, and indole-3-acetonitrile pathways can also be adopted (Duca et al., 2014; Keswani et al., 2020). IAA effect in regulating plant growth is so valuable that it has been considered a distinctive trait to test microorganisms for their potential promoting action (Etesami et al., 2015; Eida et al., 2018), and IAA-based commercial products are marketed as biostimulators (Keswani et al., 2020).

While from a plant perspective, it is certainly correct to consider PGPR positively correlated with an improvement of the overall plant status and growth performance, it is also necessary to remember that microorganisms gain proceeds from these interactions. An important inter-talk exists between plants and their associated microbes. One of the main mechanisms by which microorganisms and plants can gain a mutual advantage is driven by the release of root exudates. Root exudates are primary and secondary metabolites leaked from plant roots in their surrounding media (Bardgett et al., 2014; Vives-Peris et al., 2020). As reported in many specific reviews, microorganisms can enhance the release of root exudates, while plants have been proven to change the composition of their exudates to recruit selected beneficial microorganisms (see the example regarding ACC release reported below; Hartmann et al., 2009; Ruzzi and Aroca, 2015; Canarini et al., 2019; Vives-Peris et al., 2020; Delamare et al., 2023).

Together with the improvement of the nutritional status of the plant, microorganisms have been reported to increase plant tolerance to both biotic and abiotic stresses (Ahluwalia et al., 2021; Bessai et al., 2021; Mishra et al., 2021; Upadhyay et al., 2022). One of the bacterial traits that helps the plants grow under stress conditions is reducing the perception of the stress itself. This result is mainly achieved by altering the concentration of certain plant hormones. For example, some beneficial rhizobacteria can use 1-amino-cyclopropane-1-carboxylic acid (ACC) as a C and N source (Grover et al., 2021; Teo et al., 2022) and transform it into ammonia and α-ketobutyrate, due to the possession of the enzyme 1-aminocyclopropane-1-carboxylate deaminase (Gamalero et al., 2023). ACC is the precursor of ethylene, thus limiting its transformation to ethylene and its induced growth inhibition (Shahid et al., 2023). Plants gain proceeds from this interaction with microorganisms to the extent that ACC has been proven to be released by plants in the rhizosphere to recruit specific bacteria able to cleave ACC (Ali and Kim, 2018; Gamalero et al., 2023). Moreover, IAA also upregulates ACC synthase, the enzyme responsible for the synthesis of ACC, underlying the existence of an important interaction between the two hormones (Gamalero et al., 2023). Ethylene perception and signaling is also involved in stress tolerance and bacteria-plant interactions. Ibort et al. (2017), by analyzing the impact of two PGPR (*Bacillus megaterium* and *Enterobacter* sp. C7) on tomatoes, demonstrated that the ethylene perception is essential for the recognition of beneficial PGPR and that this perception is strain-dependent. In fact, in ethylene-insensitive plants, *B. megaterium* was recognized by the plant as a pathogen-like microorganism, leading to oxidative stress (**Supplementary Table S2**). PGPR can stimulate plant ABA production, which determines a reduction in stomatal conductance and leaf water losses under osmotic or water stress and decreases the risk of desiccation (Bresson et al., 2013; Cohen et al., 2015). On the contrary, other PGPR have been reported to induce a drop in ABA concentration after plant inoculation, thus limiting the detrimental effects of this phytohormone on stomatal conductance and growth under water stress (Kang et al., 2014; Barquero et al., 2022).

PGPR are also useful in biocontrol, preventing agricultural losses due to pathogen infections by competing with the pathogen for limiting resources, by inducing systemic responses in plants, by antibiosis, and by the synthesis of fungal cell wall lysing enzymes and pathogen-limiting volatile organic compounds (Glick et al., 2007; Oleńska et al., 2020; Grover et al., 2021). The systemic acquired resistance is associated with the activation of pathogenesis-related proteins (Wang et al., 2005; Kang et al., 2012) and the coordinated synthesis of salicylic acid. Some PGPR can also produce salicylic acid and may contribute, together with the plant-produced hormone, to increase protection against biotic and abiotic stress (Aloo et al., 2023). Cyanogenic bacteria are able to produce hydrogen cyanide (HCN), which inhibit the growth of various pathogenes, and can, therefore, be used as biopesticides in sustainable agriculture (Sehrawat et al., 2022). Pathogens like *Pythium*, *Fusarium*, or *Rhizoctonia* usually infect plants at the seedling stage. The seedling stage represents a phase of plant development characterized by a weak immune system, and plantlets are too fragile to protect themselves from pathogen infection (Feng et al., 2023). PGPR can enhance plant resistance by simply improving plant growth rate, thus shortening the stage of development in which the plant is more susceptible (Van Loon, 2007). Moreover, some bacteriocins have also been reported to stimulate plant growth (Lee et al., 2009).

In contaminated soils, several PGPR can modify the selective properties of root cell membranes to reduce the uptake of toxic compounds or to induce an accumulation of osmoprotectants (Khan et al., 2020; Gupta et al., 2022; Teo et al., 2022) while exopolysaccharides produced by rhizobacteria can provide a physical defense against root desiccation or act as a physical barrier against harmful ions (Khan et al., 2020; Mishra et al., 2021; Morcillo and Manzanera, 2021).

PGPR effect on plant responses usually induces changes in its proteomic profile. Over-production of proteins involved in ROS-reduction, photosynthetic activity, cell architecture and energy metabolism are usually observed (Rodríguez-Vázquez and Mesa-Marín, 2023). Moreover, as demonstrated by Ibort et al. (2017) in tomato plants, these changes can be strain- and plant cultivar-dependent.

Although PGPR can support the plant’s development in different ways, it is crucial to define whether the induced increase in plant biomass is due to an enhancement of photosynthesis (light and dark reactions), a reduction in C losses, or both.

## 4 Plant ecophysiology as a tool to assess plant’s perception of PGPR under non-stressed and stressed growth conditions

**Figure 1:**
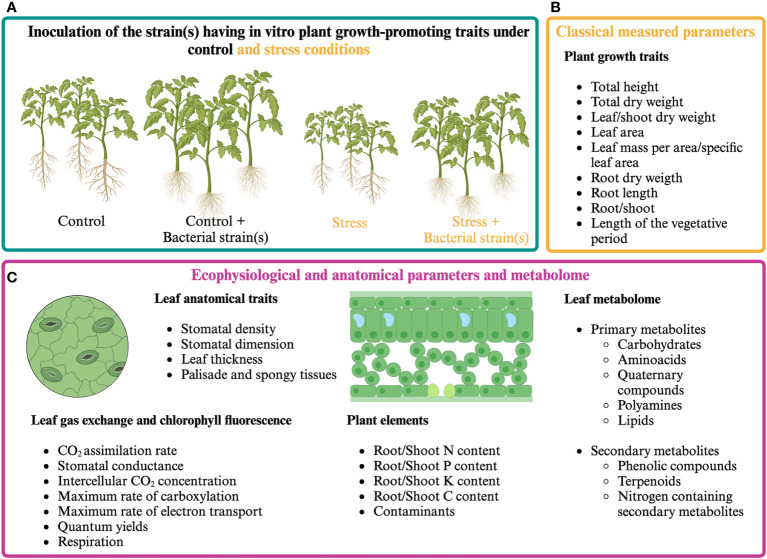
Classical and proposed multi-level approach for assessing plant responses to presumptive PGPR. **(A)** Cultivation of PGPR-inoculated and control plants under optimal and stressful growth conditions. **(B)** Classical plant growth measurements. **(C)** Proposed multi-level approach aiming at analyzing plant responses by measuring ecophysiological and anatomical parameters and analyzing plant metabolome.

The analysis of plant ecophysiological responses coupled with the quantification of morphoanatomical and growth parameters can help define how plants perceive the presence of PGPR and how they can positively affect photosynthetic activity (**Figure 1**). Plant ecophysiological responses to PGPR have been mainly studied in the model plant *Arabidopsis thaliana* and cereals, pulses, forage species, and some woody species of agronomic interest. Since 2000, many research articles have evaluated the effect of microorganisms’ application on plant growth by measuring parameters related to photosynthesis under unstressed and stressed conditions. Among these, chlorophyll fluorescence (mainly F_v_/F_m_ and ϕ_PSII_) is the most widely used, as it immediately responds to stress even before symptoms are visible on the leaf (Maxwell and Johnson, 2000; Gururani et al., 2013; Lambers and Oliveira, 2019).

F_v_/F_m_ and Ф_PSII_ have been proven to react differently to PGPR application, possibly being similar (1) or higher (2) in inoculated *vs.* control plants under stressful and non-stressful conditions; higher in inoculated *vs.* control plants under stressful conditions only (3); lower in inoculated *vs.* non-inoculated plants under stressful conditions (4) (**Table 2**; **Supplementary Table S2**). Although these parameters allow a rapid and non-destructive measure of the performance of photosystem II, neither F_v_/F_m_ nor Ф_PSII_ reflect net CO_2_ assimilation rate (Lindqvist and Bornman, 2002; for more details, see Kalaji et al., 2016), maximum RuBisCO carboxylation rate, or leaf N content (Bucher et al., 2018). Hence, chlorophyll fluorescence measurements must be coupled with leaf gas exchange determination if an understanding of the mechanisms that drive the photosynthetic and growth processes in the presence of beneficial microorganisms is desired.

The next paragraphs report a critical discussion of the main evidence regarding plant responses to PGPR. The objectives are to highlight the changes induced by beneficial bacteria on plant physiological processes under optimal growth or stress conditions to answer the following questions: is there a precise pattern of mechanisms that can be detected in plant ecophysiological responses to PGPR application? Which are the most useful parameters that should be measured to have a complete picture of the observed plant responses to PGPR? What time scale allows the detection of changes induced by PGPR on plant ecophysiological performance?

### 4.1 Effect of PGPR in non-stressed plants

#### 4.1.1 Arabidopsis thaliana

In pot experiments carried out in a growth chamber (under controlled lighting, humidity, and temperature conditions), Barriuso et al. (2008) analyzed the response of *A. thaliana* to biotic and abiotic stresses after inoculation with four different Gram-positive PGPR isolated from *Pinus* sp.

In this work, the Authors observed that the application of auxin-producing and non-auxin-producing PGPR under non-stressed conditions had different effects on the photosynthetic efficiency and fresh weight production. The application of the non-auxin-producing *Bacillus* sp. determined an increase in the F_v_/F_m_ ratio (**Table 2**) but did not affect biomass production (**Supplementary Table S1**). In contrast, applying the indole acetic acid (IAA)-producing strain *Arthrobacter oxydans* BB1 only determined an increase in fresh weight (**Supplementary Table S1**; Barriuso et al., 2008).

Works from Pare’s group on *A. thaliana* inoculated with *Bacillus subtilis* GB03 indicated a correlation between auxin-level/homeostasis and photosynthetic activity (Zhang et al., 2008; Xie et al., 2009). In cultivation experiments on Petri dishes and Magenta plant culture boxes containing half-strength Murashige & Skoog (MS) medium, the Authors demonstrated that a two-week exposure of *A. thaliana* seeding to VOCs produced by strain GB03 determined variations in the auxin level, which increased in the root tissues and decreased in the aerial portions. These changes in the auxin spatial distribution were accompanied by an increase in quantum yields of photosystem II (F_v_/F_m_ and ϕ_PSII_), chlorophyll content, and dry weight, indicating that these parameters were positively affected by both auxin production and differential transport (**Table 2**; **Supplementary Table S1**).

In pot experiments, *A. thaliana* plantlets obtained from seeds inoculated with *Phyllobacterium brassicacearum* STM196 (a PGPR isolated for its ability to antagonize the effect of high nitrate on the lateral root development; Contesto et al., 2010) showed an increase in root dry weight compared to non-inoculated controls (Bresson et al., 2013). Remarkedly, the inoculation with this PGPR did not affect *A. thaliana* shoot dry weight. The inability of the STM196 strain to produce auxin (Contesto et al., 2010) suggested that the increase in *A. thaliana* root biomass was determined by changes in the endogenous auxin homeostasis triggered by the PGPR. Interestingly, the seed inoculation with *P. brassicacearum* STM196 determined a significant increase in leaf area and number, with no change in the leaf mass (**Supplementary Table S1**) and a decrease in the CO_2_ assimilation rate compared to non-inoculated plants (**Table 2**). In inoculated plants, a shift from thicker to thinner leaves was observed, which suggested, according to Hoshino et al. (2019), STM196 induced a disturbance in the periclinal cell division and anisotropic growth. As indicated by Bresson et al. (2013) results, this effect could be determined by the increase in the sucrose content (**Supplementary Table S1**), which is known to promote paradermal growth (increase in the cell area expansion and cell division number), rather than an increase in the number of cell layers of the palisade tissue (Hoshino et al., 2019). Lobo Moreira et al. (2015) demonstrated that an increase in leaf sucrose concentration could decrease carboxylation activity and RuBisCO content, negatively affecting the CO_2_ assimilation rate. Although most studies on the effect of PGPR on plant ecophysiology and leaf gas exchange in C3 plants omit to analyze RuBisCO activity, this parameter should be carefully analyzed to understand the PGPR-plant interaction. Granot et al. (2014) reported that a decrease in CO_2_ assimilation rate can be a consequence of stomatal closure induced by an increase in ABA or sucrose concentration. The reduced transpiration and CO_2_ assimilation rate observed in *A. thaliana* inoculated with *P. brassicacearum* STM196 (**Table 2**) result from the accumulation of ABA and sucrose in shoots. At the same time, the increase in the root biomass in STM196 inoculated plants can be explained by reallocating the energy obtained, increasing the total photosynthetic area (leaf area × leaf number) and the energy use efficiency, and reducing the C losses (**Supplementary Table S1**).

#### 4.1.2 Fabaceae

As discussed for *A. thaliana*, inoculation of members of the *Fabaceae* family with PGPR differentially affects the total biomass production. Moreover, in inoculated plants, changes in the plant weight and maximum quantum yield of PSII are not linearly correlated.

In pot experiments carried out with *Glycine max* L. (soybean) inoculated with different PGPR, Algar et al. (2014) did not observe any variation in the total fresh biomass when *Pseudomonas fluorescens* N21.4, *Stenotrophomonas maltophilia* N5.18 or *Chryseobacterium balustinum* Aur9 strains were used as inoculants (**Supplementary Table S1**). In contrast, inoculation of *G. max* with *Curtobacterium* sp. M84 determined a reduction of fresh biomass production without affecting ϕ_PSII_, whose levels decreased when the previously mentioned strains were used as inoculants (**Table 2**).

Similar observations were reported by Kumari et al. (2015), who investigated the effect of *Pseudomonas* sp. AK-1 and *Bacillus* sp. SJ-5 on the growth of soybean plants. In inoculated plants, these authors observed an increase in the total root length and lateral root production compared to non-inoculated plants without any significant effect on the total fresh weight (**Supplementary Table S1**). These results highlighted the importance of the PGPR-induced modifications of the plant architecture for the correct evaluation of beneficial microorganisms that do not promote plant growth under favorable cultivation conditions.

Apart from the abovementioned studies, other findings with different *Fabaceae* species and bacterial inoculants indicate that PGPR can enhance plant growth and photosynthetic activity. In a pot experiment aiming to evaluate the effect of a consortium of three PGPRs on the germination and growth of *Trigonella foenum-grecum*, Bisht et al. (2022) reported an increase in CO_2_ assimilation rate induced by higher stomatal conductance and higher content of photosynthetic pigments (**Table 2**). The Authors showed that 45 days after the inoculation with the PGPR consortium, the increase in energy availability allowed an enhancement of leaf area, shoot length, root dry weight, and N and protein content in plant tissues of inoculated plants (**Supplementary Table S1**).

Similar positive effects of PGPRs were observed in *Cicer arietinum* inoculated with *Mesorhizobium ciceri* (diazotroph), *Serratia* sp. ST9 or SF3 (capable of solubilizing phosphate) or a combination of *M. ciceri* with the single *Serratia* sp. strains. With all the inoculants, a significant increase in the shoot, root, and nodule dry mass, root and shoot length, grain yield, and number of nodules per plant was observed (Shahzad et al., 2014; **Supplementary Table S1**). In contrast, the Authors detected higher CO_2_ assimilation rate, chlorophyll content, and grain protein content only in plants co-inoculated with *M. ciceri* and P-solubilizing *Serratia* strains (**Table 2**). Similar effects on the protein content of *C. arietinum* seeds were observed by Singh et al. (2014) and Uddin et al. (2014) utilizing phosphate solubilizing bacteria in combination with diammonium phosphate fertilization or *Rhizobium* sp. inoculation, respectively. These data indicate that for increasing the seed protein content in *Fabaceae*, a consortium of P-solubilizing and N-fixing bacteria is mandatory to reduce the use of chemical fertilization. In this respect, as shown by Shahzad et al. (2014), the measurement of the CO_2_ assimilation rate can be a rapid tool to predict the efficiency of microbial consortium on the increase in seed quality.

An enhancement of the grain yield in response to the application of a consortium of two *Bacillus* strains (*B. pumilus* S4, with phosphate solubilization and siderophore production capacity, and *B. mycoides* S7, an IAA producer) was also observed in a field experiment on *Phaseolus coccineus* (Stefan et al., 2013; **Supplementary Table S1**). The experimental design of the work included a non-inoculated control, a treatment in which the plants were inoculated with the microbial consortium S4+S7, and two additional treatments in which *P. coccineus* was inoculated, separately, with the single *Bacillus* strains (*B. pumilus* S4; which did not increase grain yield; *B. mycoides* S7, which increased grain yield to a less extent). This study highlighted that the increase in assimilation rate due to the application of PGPR might not be immediate: the increases in the CO_2_ assimilation rate in S7 and S7+S4 and water use efficiency were observed during the first vegetative (28 days after inoculation) and at early flowering (42 days after inoculation) stages (**Table 2**). As chlorophyll content did not change over time, the results reported by Stefan et al. (2013) suggest that applying the *Bacillus* strains determined an increase in the electron transport efficiency or carboxylation rate. Although the final grain yield at harvest (96 days after inoculation) was higher in inoculated plants, the CO_2_ assimilation rate did not differ among treatments (**Table 2**). The latter scenario highlights the importance of deciding the timing of measurements, as the determination of photosynthetic and growth parameters at the end of the plant agronomical cycle may not reflect the actual changes in plant responses triggered by microorganisms.

#### 4.1.3 Graminaceae

Inoculation with the Gram-negative endophyte PGPR *Burkholderia phytopfirmans* PsJN, an IAA and ACC deaminase producer, positively affects the plant growth of different *Graminaceae* species. In *Panicum virgatum*, the endophyte stimulates root length, diameter, area, and dry weight, leaf elongation, leaf biomass, leaf area, specific leaf weight (dry weight of leaves/leaf area) while decreasing specific root length (root length/root dry mass), indicating changes in development (Wang et al., 2015; **Supplementary Table S1**). In this study, *B. phytofirmans* strain PsJN has also been demonstrated to increase CO_2_ assimilation rate and aboveground biomass 17 days after switchgrass seedling inoculation, initially without increased stomatal conductance (**Table 2**). Therefore, the enhanced CO_2_ assimilation rate was dependent on an increase in carboxylation activity rather than stomatal conductance, as suggested by the lower intercellular CO_2_ concentration observed in inoculated plants compared to non-bacterized ones (**Table 2**). Developing a more efficient root apparatus stimulated by the auxin-producer PGPR might have improved nutrient uptake, increasing photosynthetic rates.

In two *Zea mays* cultivars (Mazurka and Kaleo), PsJN stimulation of CO_2_ assimilation rate (+45% in Mazurka, + 25% in Kaleo) was accompanied by enhanced stomatal conductance and quantum yield of PSII in dark-adapted leaves (Naveed et al., 2014; **Table 2**). The superior photosynthetic activity increased plant above and below-ground biomass (+47.8% in Mazurka, +28.7% in Kaleo), augmenting leaf number and area. In the same experiment, although the PGPR *Enterobacter* sp. FD17 increased assimilation rate (+30% in Mazurka, + 19% in Kaleo) and total biomass production (+28.7% in Mazurka, +16.4% in Kaleo), F_v_/F_m_, stomatal conductance and leaf number were not enhanced by the microorganism, highlighting a different PGP activity compared to PsJN (**Table 2**). This might be due to the better *Burkholderia* colonization ability observed with PsJN by Naveed et al. (2014).

A contrasting effect was observed in a greenhouse experiment on the same plant species inoculated with *Bacillus megaterium*; leaf gas exchanges were not affected by the treatment, and shoot biomass did not increase, while root biomass decreased upon inoculation (Romero-Munar et al., 2023; **Table 2**). On the other hand, according to the observations made by Stefan et al. (2013) in *P. coccineus*, inoculation of *Triticum aestivum* with a consortium of PGPR (*Bacillus* sp. and two Azospirilli) increased plant yield and rose N, P and potassium (K) content in grains, suggesting similar induced microbial mechanisms (Akhtar et al., 2021; **Supplementary Table S1**).

#### 4.1.4 Other plant species

##### 4.1.4.1 Tree species

PGPR application in tree species has emerged as an important strategy to improve seedling acclimation (Nunes Tiepo et al., 2018).

Inoculation of 1-year-old *Sambucus williamsii* seedlings with *Acinetobacter calcoaceticus* X128 determined an increase in root biomass, CO_2_ assimilation rate, and stomatal conductance (Liu et al., 2019b; **Table 2**). Interestingly, a higher ABA content was only detected in root tissues in inoculated plants, which could be why the increase in the ABA content failed to induce stomatal closure as expected. The inoculation with the X128 strain also determined an increase in the shoot cytokinin content compared to non-inoculated plants. The inhibitory effects of cytokinins and auxins on ABA stomatal closure induction were already observed by Tanaka et al. (2006), who reported that both hormones modulate ethylene biosynthesis, but only cytokinins inhibit the ABA-induced reduction of osmotic pressure in the guard cells.

Differently from what was observed in *S. williamsii*, inoculation of *Trema micrantha* and *Cariniana estrellensis* (two neotropical tree species) with *Azospirillum brasilense* Ab-V5, *Bacillus* sp., and *Azomonas* sp. did not determine any increase in the assimilation rate (Nunes Tiepo et al., 2018; **Table 2**). In contrast, inoculation of *C. estrellensis* with *Azorhizophillus* sp. determined reduced CO_2_ assimilation rate and stomatal conductance and increased H_2_O_2_ leaf concentration (**Table 2**). These variations can be connected to oxidative stress that did not affect biomass production.

##### 4.1.4.2 Vitis vinifera

The Ait Barka group at the University of Reims observed contrasting effects on the leaf gas exchanges of *Vitis vinifera* cultivar Chardonnay after inoculation with *Burkholderia phytofirmans* PsJN (**Table 2**). In preliminary work, in which the micro-propagated plants were treated with a diluted PsJN cell suspension (10^6^ CFU ml^-1^), Ait Barka et al. (2006) detected an increase in the assimilation rate and plant growth compared to the non-inoculated plants (**Table 2**). In a subsequent paper, the same research group performed a similar experiment using a 100-fold more concentrated bacterial inoculum (10^8^ CFU ml^-1^). In the latter conditions, the lowest CO_2_ assimilation rate was observed in the PsJN-inoculated plants (Fernandez et al., 2012; **Table 2**). These results highlight the importance of testing different inoculum concentrations to evaluate the effect of a PGPR on the target plant system. At the same time, the decrease in the CO_2_ assimilation rate determined by an increase in the PsJN cell density indicated that, at high cell dosages, a PGPR could be perceived by the plant as an external agent causing biotic stress.

Backes et al. (2021) also reported a similar effect in barley inoculated with a different strain belonging to the same genus: *Burkholderia* sp. strain B25. The foliar application of strain B25 at a concentration of 10^9^ CFU ml^-1^ was associated with increased cyclic electron flow (**Table 2**). This pathway allows the synthesis of ATP and is involved in protecting PSI in plants under stress. Under this point of view, the measurement of the plant ecophysiological responses to PGPRs can be a valuable tool to optimize the dose of the inoculum and the application mode of the PGPR biostimulant (seed, soil or foliar; Jing et al., 2023).

##### 4.1.4.3 Salicornia ramosissima

The effect of the PGPR consortium of *Thalassaspira australica* SRT8, *Pseudarthrobacter oxydans* SRT15, and *Vibrio neocaledonicus* SRT1 was analyzed by Mesa-Marín et al. (2020). The application of microorganisms did not enhance CO_2_ assimilation rate or stomatal conductance, and the quantum yield of PSII in dark-adapted leaves was lower, while dissipated energy flux was higher compared to non-inoculated plants, indicating a reduced amount of energy transfer to photochemistry reactions (**Table 2**). Nevertheless, the relative growth rate increased in inoculated *S. ramosissima*, probably because of reduced C losses.

### 4.2 Effect of PGPR under abiotic and biotic stress

#### 4.2.1 Salinity

PGPR have been shown to affect leaf gas exchanges under saline conditions positively. This can be achieved by increasing stomatal conductance, synthesizing secondary metabolites and antioxidant enzymes, or by altering the selectivity of ion absorption in the roots. Particularly, Wang et al. (2023) proved that the application of *Bacillus pumilus* JIZ13 on *Oryza sativa* exposed to high salinity (NaCl 300 mM) increased assimilation rate, stomatal conductance, and chlorophyll content compared to non-inoculated plants (**Supplementary Table S2**). The ability of the JIZ13 strain to produce IAA and siderophores and solubilize phosphate also determined an increase in nutrient acquisition and root growth. The increased production of compatible solutes (i.e., proline and sugars) and antioxidant enzymes could be supported by higher energy availability associated with increased photosynthetic activity. The accumulation of osmolytes and enzymatic activities were responsible for a decrease in oxidative stress by regulating the steady-state concentration of ROS (malondeadehyde, H_2_O_2_, and O_2_^-^).

Similar results on the photosynthetic activity were obtained by Bisht et al. (2022) in fenugreek plants grown under moderate salinity (70 and 150 mM NaCl) after inoculation with a microbial consortium including *Azotobacter chroococcum*, *Enterobacter asburiae*, and *Lactococcus lactis*. Also, in this PGPR-plant interaction, the increased photosynthetic rate was coupled with higher stomatal conductance, transpiration, intercellular CO_2_ concentration, chlorophyll *a* and total chlorophyll, and carotenoids, compared to non-inoculated plant, with a consequent increase in shoot and root dry weight (**Supplementary Table S2**).

A positive effect of PGPR application on photosynthetic rates, chlorophyll content (*a* and *b*), and stomatal conductance was also observed inoculating *Glomus versiforme* with *Micrococcus yunnanensis* (Afrangan et al., 2023; **Supplementary Table S2**). The Authors observed that, under all salinity levels, the PGPR application determined no changes in Na^+^ concentration in root and shoots compared to non-inoculated plants and an increase in K^+^ in roots and shoots, probably related to changes in selective ion absorption and transportation (**Supplementary Table S2**). In plants treated with *M.yunnanensis*, the improved redox status was accompanied by increased antioxidant enzymatic activities and carotenoid concentrations similar to what was observed with *B. pumilus* JIZ13 in *O. sativa*.

In *Glycine max* grown under saline stress, Kumari et al. (2015) observed that the inoculation with *Pseudomonas* sp. strain AK-1 and *Bacillus* sp. determined an increase in the total chlorophyll content compared to non-inoculated plants (**Supplementary Table S2**). Under the same conditions, the PGPR determined an enhancement in plant biomass and the production of molecules (proline and lipoxygenase), which can be involved in determining salt tolerance (**Supplementary Table S2**).

García-Cristobal et al. (2015), studying the response of *O. sativa* to two non-nodulating diazotrophic bacteria (*Bacillus* sp. L81 and *Aeromonas* sp. AMG272) under saline conditions, observed a reduction of the stress symptoms, which was not accompanied by significant variations in the efficiency of photosystem II (**Supplementary Table S2**). The relief of saline stress was coupled to a variation in enzymatic activities related to oxidative stress, especially ascorbate peroxidase and superoxide dismutase (**Supplementary Table S2**).

#### 4.2.2 Drought

Under drought stress, the application of PGPR has shown different effects on plant growth and photosynthetic efficiency.

In pot culture experiments carried out with *Sambucus williamsii* grown under a reduced water regime, Liu et al. (2019b) demonstrated that inoculation with *Acinetobacter calcoaceticus* X128 determined a delay in the inhibitory effect of drought stress (**Supplementary Table S2**). Under the same cultivation conditions, non-inoculated drought-stressed plants showed a detectable reduction of stomatal conductance and assimilation rate already on day six from the onset of the treatment. In inoculated plants, the reduction in stomatal conductance and assimilation rate were less pronounced compared to non-inoculated plants. Both parameters remained significantly higher after long-term exposure to drought stress (days 24 and 30), supporting the conclusion that the crosstalk between *A. calcoaceticus* X128 and the plant triggered a response that mitigates the drought symptoms. The Authors correlated the lower intercellular CO_2_ concentration measured in inoculated plants at the end of the treatment (day 36) to an increase in the carboxylative activity by RuBisCO induced by the PGPR. Moreover, X128 inoculated plants showed a complete recovery of the photosynthetic activity 6 days after rewetting: assimilation rate and stomatal conductance were similar to the control and higher than non-inoculated plants.

The same Authors, analyzing the protecting effect of *A. calcoaceticus* X128 on *S. williamsii* under different severity levels of drought stress (from light to severe), showed that this strain, in addition to the increase in the assimilation rate, stomatal conductance, and dry mass, determined an increase in the relative water content of the plant under all drought conditions (**Supplementary Table S2**; Liu et al., 2019a).

An increase in the photosynthetic activity (higher assimilation rate and stomatal conductance, higher chlorophyll *a* and *b* content) was also reported by Akhtar et al. (2021) in *T. aestivum* subjected to drought treatment and inoculated with a consortium of one *Bacillus* sp. and two *Azospirillum* strains (**Supplementary Table S2**). The Authors observed that the enhancement of the photosynthetic activity was related to an increase in some antioxidant enzymatic activities (peroxidase and catalase) and the concentration of K and P in root and shoot tissues. Moreover, N, K, and P content in grains was ameliorated, increasing the nutritional value of the production (**Supplementary Table S2**). Although the CO_2_ assimilation rate was higher in inoculated plants, no biomass increase was observed, probably because of the high energy costs of secondary metabolite production.

In an interesting study on the effect of two endophytes (*Burkholderia phytofirmans* PsJN and *Enterobacter* sp. FD17) on the growth of two maize cultivars (Mazurka and Kaleo) under drought conditions, Naveed et al. (2014) reported that the inoculation with both PGPR determined greater assimilation rate, higher chlorophyll content and F_v_/F_m_ compared to non-inoculated plants (**Supplementary Table S2**). In PsJN-treated plants, the increase of these parameters was associated with higher stomatal conductance, while no effect on stomatal conductance was observed in FD17-inoculated plants (**Supplementary Table S2**). Data reported by Naveed et al. (2014) also indicated that the assimilation rate measured in PsJN-inoculated plants under drought stress was similar to that in non-stressed plants (**Supplementary Table S2**). At the same time, the inoculation of this microorganism allowed the authors to obtain an increase in the aboveground biomass even compared to non-stress plants. The latter results were also observed for FD17 but only in one of the two cultivars (Mazurka; **Supplementary Table S2**).

Inoculation of *C. arietinum* seeds with strains belonging to *M. ciceri* (MC), *S. marcescens* SF3, and *Serratia* sp. ST9 determined increased plant biomass, height, and grain yield without significantly enhancing the photosynthetic parameters (Shahzad et al., 2014; **Supplementary Table S2**). Under the same experimental conditions, an inoculation of *C. arietinum* seeds with a combination of MC and the other strains (MC+SF3 and MC+ST9) determined both an increase in the plant growth and the photosynthetic parameters (**Supplementary Table S2**). These results indicate that a correct and comprehensive measurement of the photosynthetic parameter can be valuable to studying PGPR-plant interactions and the cumulative effect of combinations of selected PGPR strains on plant physiology. Differently from what Naveed et al. (2014) observed, PGPR application might also not ameliorate growth conditions under stress. Inoculation of the tree species *Trema micrantha* and *Cariniana estrellensis* with *Bacillus* sp., *Azomonas* sp., and *Azorhizophillus* sp. grown under drought conditions did not increase plant biomass or assimilation rate compared to non-inoculated plants (Nunes Tiepo et al., 2018; **Supplementary Table S2**). Only *Azospirillum brasilense* Ab-V5 provoked a positive effect on photosynthetic rates in *C. estrellensis*, which were induced by a higher carboxylation efficiency rather than enhanced stomatal conductance (**Supplementary Table S2**). Moreover, Romero-Munar et al. (2023) also found no effect on assimilation rate, stomatal conductance, root and shoot biomass in maize treated with *B. megaterium* and subjected to the combined drought and high-temperature stresses compared to non-inoculated plants (**Supplementary Table S2**). Although changes in hormone concentrations were observed, these were insufficient to induce a positive effect on plant growth (**Supplementary Table S2**). Different responses to drought were observed inoculating recombinant tomato’s inbred lines (RIL20, 40, 66, 100) with the PGPR *Variovorax paradoxus* 5C-2 (**Table 2**; **Supplementary Table S2**; Calvo-Polanco et al., 2016). Only in one of the four inbred lines (RIL66), the PGPR determined an increase in the aboveground biomass. Interestingly, this effect was not accompanied by a concomitant increase in CO_2_ assimilation rates and by a decrease in the proline content and the abundance of plasma membrane intrinsic proteins subfamily PIP1 and PIP2 (aquaporin channels that facilitate the passive movement of water molecules from cell to cell). These results, together with the negative observed correlation between root hydraulic conductivity and phosphorylated PIP2 in RIL66 demonstrated that the enhanced root hydraulic conductivity in inoculated plants was related to altered apoplastic water flow. This study highlights the importance of using selected plant material which positively respond to PGPR inoculation to ameliorate the cultivation conditions under drought stress.

In *A. thaliana* inoculated with *P. brassicacearum* STM196, a reduction in assimilation rate and stomatal conductance (transpiration) (**Supplementary Table S3**), not coupled with a decrease in stomatal density were observed by Bresson et al. (2013) under drought stress. The reduction in stomatal conductance triggered by an increase in ABA content limited CO_2_ assimilation but protected the plant against desiccation by increasing water use efficiency, leaf water preservation (relative water content), and drought resistance (**Supplementary Table S2**).

#### 4.2.3 Flooding

Waterlogging and flooding trigger a series of biological, chemical, and physical modifications in soils which can limit plant growth and survival. The supply of oxygen to the roots becomes one of the main factors reducing plant growth in these environments. Hormonal signals transmitted from the roots to the shoots (ABA and cytokinin) usually induce stomatal closure, limiting leaf gas exchange (Koslowski, 1997), while, in tolerant plants, aerenchyma and adventitious root formation are usually observed (Visser et al., 1995; Blom and Voesenek, 1996; Abou Jaoudé et al., 2013). Together with plant responses, PGPB have also been reported to induce plant anatomical modifications aiming at reducing root anoxia. For example, Ueckert et al. (1990) demonstrated that inoculation with *Azospirillum brasilense* increased the size of aerenchyma and, proportionally, the oxygen concentration in the rhizosphere. In the same study, an interesting increase in the permeability of the cell wall was observed which increased the oxidation of the rhizosphere.

Salazar-Garcia et al. (2022) reported an increase in stomatal conductance, chlorophyll index and optimal F_v_/F_m_ values in radish plants subjected to flooding and treated with *Azospirillum brasilense*, compared to non-inoculated plants (**Supplementary Table S2**). The overall betterment of the photosynthetic performances was also remarkable in terms of plant architecture and biomass production: the leaf area, leaf number, and diameter of the tuberous roots were all comparable to plants grown under control conditions, while the total plant dry weight, although lower compared to control plants, was significantly higher than non-inoculated waterlogged ones. However, the mechanisms behind this growth enhancement remained unclear. In fact, despite *A. brasilense* being a well-studied PGPR known for its multi- (fixing nitrogen, production of IAA, gibberellins, ABA, cytokinins and ethylene), and some strain-specific promoting activity, its growth promotion is often the result of cumulative effects (Bashan and de-Bashan, 2010).

Czarnes et al. (2020), evaluating the effect of *Azospirillum lipoferum* CTR1 inoculation on maize subjected to waterlogging, obtained contrasting results analyzing different cultivars. Inoculation with strain CTR1 enhanced the photosynthetic performances of the cultivar FriedriXX, with a slight increase in the root length and a decrease in the leaf area compared to non-inoculated plants. In contrast, inoculation of cultivar FuturiXX with *A. lipoferum* CTR1 determined a reduction in the Ф_PSII_ and photosynthetic rate.

Thus, although using bacteria with multiple promoting traits like *Azospirillum* strains might be advantageous in terms of growth stimulation, experiments need to be designed more accurately taking into account both the cultivar physiological performances and the strain promoting traits if a clear understanding of the specific mechanism(s) taking part to the growth stimulation process needs to be achieved.

#### 4.2.4 Chilling

Chilling, i.e., the cold temperature above the freezing point (0-15°C), inhibits plant growth and development in susceptible plants, inactivating plasma membrane and tonoplast, decreasing photosynthesis because of increased stomatal resistance and photosystems’ damage, causing metabolic disorders (Larcher, 1995). When chilling occurs at a high light intensity, oxidative stress and photoinhibition were observed: the accumulation of ROS can lead to programmed cell death, a strategy plants use to escape oxidative damage (Aslam et al., 2022).

The effect of PGPRs on cold stress was studied in monocotyledons and dicotyledons using different bacterial systems. In works carried out at the University of Reims with micro-propagated grapevine (*Vitis vinifera*) cultivar Chardonnay, the research group headed by Ait Barka demonstrated that inoculation with *Burkholderia phytofirmans* PsJN induced physiological changes that enhanced the adaptation of plants to cold stress (**Supplementary Table S2**). In particular, the inoculation with strain PsJN determined increased CO_2_ fixation and O_2_ evolution and starch and proline content, indicating an osmotic adjustment (Ait Barka et al., 2006; **Supplementary Table S2**). Moreover, the conversion of fructose and mannose in ascorbic acid might have induced the stimulation of ROS-scavenging via ascorbate synthesis, reducing oxidative stress compared to non-inoculated grapevine plantlets (Fernandez et al., 2012).

#### 4.2.5 Nutrient limitations

The role of PGPR in contrasting the adverse effects of nutrient limitations on plant growth has been widely reviewed, especially in poor, arid, and saline soils (Poria et al., 2022; Khan et al., 2023; Naorem et al., 2023). However, studies aiming at analyzing the effect of bacteria with specific abilities to enhance plant nutrient status on photosynthesis are limited.

An interesting study was conducted by Calzavara et al. (2018), who analyzed the effect of applying two PGPR to maize plants grown under optimal and reduced N fertilization (**Table 3**). Under low nitrogen input, the two microorganisms, a diazotroph *A. brasilense* (Ab-V5) and a no-nitrogen-fixing *Bacillus* sp. strain (ZK), affected physiological plant performances differently without inducing a modification in plant growth compared to non-inoculated plants. Inoculation with *Bacillus* sp. ZK determined an increase in CO_2_ assimilation rates and maximum quantum yield of PSII without affecting plant biomass and architecture or the leaf content of organic and inorganic N compounds compared to non-inoculated plants.

**Table 3:** Effects of PGPR inoculation on photosynthetic parameters, anatomical traits, nutrients’ content, and metabolites measured in plants grown under low or high N, or with the addition of an insoluble P source (rock phosphate) compared to non-inoculated plants (↑: increase; ↓: decrease; = non-significant variations).

Plant species	Plant Growth-Promoting strain	Nutrient treatment	Parameter	PGPB vs. Non-inoculated	Reference
*Avicennia marina*	*Oceanobacillus picturae* ^k,m^	Rock phosphate	Available P	↑	El-Tarabily and Youssef, 2010
			No. Branches	↑	
			Root length	↑	
			Shoot dry mass	↑	
			Root dry mass	↑	
			Leaf area	↑	
			Root and shoot N,P,K,S,Mg,Fe,Zn,Cu	↑	
			Stem circumference	↑	
			No. Xylem vessels	↑	
			Mean xylem vessel diameter	↑	
			V_cmax_	↑	
			J_max_	↑	
			V_TPU_	**↑**	
*Cucumis sativus*	*Streptomyces* HM2^h,k,m^ Streptomyces HM3^h,k,m^ *Streptomyces* HM8^h,k,m^ *Streptomyces bicolor* HM10^h,k,m^ Consortium of HM2, HM3, HM8, HM10	Rock phosphate	Plant height	↑	Omar et al. (2022)
			No. Leaves	↑	
			Root length	= HM2,10,cons.	
				↑ HM3,8	
			Leaf dry mass	= HM3,8,10,cons.	
				↑ HM2	
			Stem dry mass	**=**	
			Root dry mass	**=**	
			Leaf area	↑	
			CO_2_ assimilation rates	**=**	
			Relative growth rate	**=**	
			Fruit length	= HM2,8,10,cons.	
				↑ HM3	
			Fruit diameter	= HM10	
				↑ HM2,3,8,cons.	
			No. Fruits	↑	
			Fruit fresh weight	↑	
			Fruit firmness	**=**	
			P in soil	= HM2	
				↑ HM3,8	
				↓ HM10,cons.	
			P in plants	= HM2,10,cons.	
				↑ HM3,8	
*Zea mays*	*Bacillus* sp. ZK^h^ *Azospirillum brasilense* Ab-V5^h,j^	Low N (LN) High N (HN)	Epidermis thickness	=	Calzavara et al., 2018
			Cortex	=	
			Central cylinder	↑ ZK	
				= Ab-V5	
			Metaxylem vessel element area	↑ ZK	
				= Ab-V5	
			Metaxylem vessel element number	↑	
			Istantaneous carboxylation efficiency	= LN	
				= HN ZK	
				↑ HN Ab-V5	
			F_v_/F_m_	↑ LN ZK	
				= LN Ab-V5	
				= HN	
			CO_2_ assimilation rates	↑ LN ZK	
				= LN Ab-V5	
				= HN ZK	
				↑ HN Ab-V5	
			Root length	= LN	
				↓ HN	
			Shoot length	= LN	
				= HN ZK	
				↑ HN Ab-V5	
			Root dry mass	= LN	
				↑ HN ZK	
				= HN Ab-V5	
			Shoot dry mass	= LN	
				↑ HN ZK	
				= HN Ab-V5	
			Nitrate reductase activity	= LN	
				↓ HN ZK	
				= HN Ab-V5	
			Leaf nitrate	= LN	
				= HN ZK	
				↑ HN Ab-V5	
			Leaf ammonium	= LN ZK	
				↑ LN Ab-V5	
				= HN	
			Leaf amino acids	= LN ZK	
				↑ LN Ab-V5	
				=HN	
			Leaf proteins	= LN	
				↓ HN	
			Total soluble sugars	= LN ZK	
				↑ LN Ab-V5	
				= HN ZK	
				↓ HN Ab-V5	

*Electron transport energy flux.

a ACC deaminase.

b Biocontrol.

c Biofilm formation.

d Chitinases production.

e Cytokinins production.

f Exopolysaccharide production.

g Glucose/ABA sensing.

h IAA production.

i Induction of systemic resistance.

j N fixation.

k Phosphate solubilization.

l Phytase production.

m Siderophore production.

n VOCs production.

°Cd tolerance.

p ABA production.

q Osmolyte production.

r Effect on root architechture.

s Effect on root hydraulic properties.

Conversely, no variations in the photosynthetic performances, plant architecture and plant biomass were induced by *A. brasilense* Ab-V5 compared to non-inoculated plants. Still, leaf N compounds significantly increased. The scenario changed under high N input: in plants inoculated with the diazotroph *A. brasilense* strain Ab-V5, CO_2_ assimilation rates were higher due to increased carboxylation activity. However, no increase in plant biomass occurred compared to non-inoculated plants. In plants inoculated with *Bacillus* sp. strain ZK, the higher N availability did not increase CO_2_ assimilation rates, although an increase in above- and belowground biomass was observed. Unexpectedly, in this study, the diazotroph *A. brasilense* strain Ab-V5 did not produce an increase in CO_2_ assimilation rates or biomass under low N input, but the opposite occurred. These data indicate that an increase in the soluble sugar concentration in the leaves have a negative effect the photosynthetic process. In fact, under low N inputs, total soluble sugar concentration increased in the leaves, and vice versa when the input on N was high. Again, as already reported, changes in sugar metabolism induced by microorganisms might be important in affecting plant photosynthesis. Nevertheless, in this experiment, the observed increase in assimilation rates never produced an increment in plant biomass, independently of N availability, demonstrating that other extra carbon and energy were unavailable for plant growth. The authors hypothesized that bacterial signals might have increased rhizodeposition to support the plant–bacteria mutualism.

Regarding phosphorus limitations, both Gram-positive and Gram-negative bacteria with phosphate solubilizing activity have been proven to beneficially affect plant photosynthesis, growth, or yield when the inoculated plants were supplied with insoluble P (**Table 3**).

Omar et al. (2022) showed that inoculation of cucumber plants with four *Streptomyces*-affiliated strains having the ability to solubilize phosphate and produce IAA and siderophores had a positive effect on plant height, leaf number, area, fruit number, and fresh weight compared when rock phosphate was used as P fertilizer. Nevertheless, *Streptomyces* strains stimulated plant growth to different extents, independently of the observed *in vitro* quantified promoting traits. Notably, strains having similar P-solubilizing abilities (HM2, HM8 and HM10) differently affected plant growth performances (leaf number, plant height and root length) and fruit production. On the other hand, inoculation of cucumber plants with *Streptomyces* strains having significantly different P-solubilizing activity (HM3 and HM8) determined a similar stimulatory effect on plant growth and fruit production. These data indicate that a high P-solubilizing potential determined under laboratory conditions is not always associated with a superior plant growth promotion in the presence of water-insoluble P.

Similar evidence was obtained by Collavino et al. (2010) analyzing the effect of two strains with high P-solubilizing activity, *Enterobacter aerogenes* R4M-A and *Burkholderia* spp. R4M-F, on *Phaseolus vulgaris*. The authors reported an increase in shoot and root dry mass, leaf area, P concentration and CO_2_ assimilation rates only in plants inoculated with *E. aerogenes* R4M-A.

Omar et al. (2022) also reported that the biomass increase observed in *Streptomyces*-inoculated cucumbers was not associated with higher CO_2_ assimilation rates, indicating that the interaction between this PGPR and the plant might have reduced carbon loss (**Table 3**).

To date, only few studies have been conducted to assess the effect of PGPR inoculation on plant carbon losses, through respiration or exudation. Regarding losses through respiration, Vonderwell et al. (2001) observed a decrease in root respiration coupled with an increase in total root length, root and shoot biomass and IAA root concentration in loblolly pine six weeks after inoculation with the bacterial strain INR7. On the other hand, C losses through increased exudation have been reported after inoculation of *Cupressus sempervirens* with *Bacillus subtilis* and *Pseudomonas stutzeri* (Oppenheimer-Shaanan et al., 2022), sorghum seedling with the nitrogen-fixing bacteria *Azospirillum brasilense*, *Azotobacter vinelandii* or *Klebsiella pneumoniae* (Lee and Gaskins, 1982).

The use of consortia of P-solubilizing bacteria provide a more complex picture of the PGPR-plant interaction in low-phosphate conditions. Using two-month-old pot transplanted strawberry (*Fragariavesca* var. Rociera) plants and a consortium of five PGPR (SDT3, HPJ40, SMT38, SRT15 and S110, respectively affiliated to *Pseudomonas* sp, *Bacillus zhangzhouensi*, *Bacillus velezensis*, *Pseudarthrobacter oxydans*, and *Variovorax*), Valle-Romero et al. (2023) analyzed the impact of the bacterial inoculation on plants grown under sub-optimal phosphorous fertilization (**Table 3**). The bacterial consortium included three P-solubilizing strains (SDT3, HPJ40, SRT15) and 2 non-P-solubilizing bacteria (SMT38 and S110) producing ACC-deaminase activity. Four of these five strain were able to fix nitrogen (HPJ40, SMT38, SRT15 and S110) and produce siderophores (SDT3, HPJ40, SMT38 and S110); two of them also were able to produce IAA under laboratory conditions (SRT15 and S110). Under phosphorous limitation or in the presence of water-insoluble P, the plant inoculation with the PGPR consortium had a positive effect on CO_2_ assimilation rates, stomatal conductance, carboxylation activity by RuBisCO and water use efficiency compared to the non-inoculated plants. However, root and shoot biomass only significantly increased when plants were grown with insoluble P. Decoupling of photosynthesis and plant growth in inoculated plants due to the lack of insoluble P addition were mainly explained by an invariant mesophyll conductance, and a less marked increase in V_cmax_, Φ_PSII_ and electron transport rate in the short-term compared to bio-fertilized and insoluble P-treated plants. Moreover, P and potassium (K) leaf and root concentrations, and C/N were higher in all inoculated plants, and even higher when plants were supplied with insoluble P.

These experiments clearly demonstrate that an amelioration of the plant photosynthetic performances might not necessarily be translated into a biomass increase (and vice versa). Considering that PGP activity positively and negatively influence the plant carbon balance, the amelioration effects associated to PGPR inoculation should be evaluated integrating the analysis of photosynthetic activity, plant biomass production and plant architecture.

#### 4.2.6 Heavy-metals and pollutants

Plant toxicity by heavy metals has emerged as one of the most severe threats to crop production (Riyazuddin et al., 2022). Heavy metals have been reported to induce modifications in the structure and function of stomata (Guo et al., 2023) and RuBisCO activity (Li et al., 2010; Dhir et al., 2011) and altering the functionality of photosystems, thus negatively affecting light and dark reactions of photosynthesis (Riyazuddin et al., 2022).

Inoculation with PGPR has been shown to reduce the effect of toxicity of these pollutants in plants. For example, a reduction of metal ion (Zn, Cd) transfer from the roots to the shoots and a higher As accumulation in roots but not in the leaves were reported after inoculation of *Burkholderia* sp. D54 on *Lolium multiflorum* seeds and plantlet roots grown in heavy-metal contaminated paddy soil (200 mg kg^-1^ for Zn, 30 mg kg^-1^ for As, 0.3 mg kg^-1^ for Cd and 80 mg kg^-1^ for Pb; Guo et al., 2014; **Supplementary Table S2**). Although photosynthetic rates did not increase in response to inoculation, both root and shoot biomass were significantly higher compared to non-inoculated plants, and this did not decrease the total plant bioaccumulation (**Supplementary Table S2**). Inoculation of *L. multiflorum* and *G. max* with *Bradyrhizobium* sp. YL-6 showed contrasting effects when plants were grown in soil with 20 mg kg^-1^ Cd (Guo and Chi, 2014). Inoculation increased chlorophyll and carotenoid content in both species, though an enhancement in shoot biomass was observed only in *L. multiflorum* (**Supplementary Table S2**). The Cd root concentration in this plant was less than half compared to *G. max* (**Supplementary Table S2**). The higher Cd concentration in the roots seemed to have limited aboveground biomass production in this last species. The root/shoot being almost 80% lower than *L. multiflorum* (0.311 and 0.06, respectively, for *L. multiflorum* and *G. max* under drought). As Mg and Fe concentrations were higher, while Cd was lower in *G. max* leaves compared to *L. multiflorum*, we can hypothesize that the lack of an increase in above and belowground biomass was not the result of a decreased availability of these two ions (**Supplementary Table S2**). Belowground biomass allocation seems, therefore, to play an important role in determining the positive effect of PGPR under heavy metal stress.

A different scenario was reported by Mesa-Marín et al. (2020) in *Salicornia ramosissima* subjected to heavy-metal pollution and inoculated with *Thalassaspira australica* SRT8, *Pseudarthrobacter oxydans* SRT15 and *Vibrio neocaledonicus* SRT1. The three PGPR enhanced relative growth rate and stem branches increment as a result of a higher photosynthetic rate, which was due to an increase in RuBisCO activity rather than higher stomatal conductance, as suggested by the lower CO_2_ intercellular concentration (**Supplementary Table S2**). Inoculation also enhanced the quantum yield of PSII and decreased the dissipation energy flux (**Supplementary Table S2**). As Cu, Ni, Pb, and Zn concentrations were higher in inoculated plants compared to non-inoculated ones, a positive effect of the treatment might have been connected to an optimization of metal compartmentalization or an increase in antioxidant activity, which, however, was not measured in this work (**Supplementary Table S2**).

Another important class of pollutants that negatively affect plant physiology and morphology is represented by petroleum hydrocarbons (PHs). Soil contamination by PHs determines a reduction in plant growth associated with an ethylene response and oxidative stress in the root tissues.

In *Secale cereale* cultivated in PHs contaminated soil, Gurska et al. (2015) reported that inoculation with *Pseudomonas putida* UW4, an IAA and ACC deaminase producing PGPR, determined an increase in the root diameter compared to non-inoculated plants. This morphological modification was coupled to a decrease in non-photochemical quenching (heat dissipation) and an increase in the expression of genes involved in the defense/stress response. PDS (encoding phytoene desaturase, a key enzyme involved in synthesizing carotenoids), PABP (encoding an RNA-binding protein important for mRNA translation and metabolism), cytochrome P450 monooxygenases (which catalyze many reactions involved in metabolism contaminants, increasing solubility and bioremediation), and PMA (encoding the plasma membrane H^+^ATPase, that is involved in inducing stomatal opening, and regulating cell elongation and intracellular pH) were all upregulated in shoots of UW4 inoculated plants.

In maize grown in soil contaminated with spent metalworking fluids (MWFs) inoculated with *P. fluorescens* Aur6 determined a significant increase in the Hill reaction and the number of chloroplasts compared to non-inoculated plants (Grijalbo et al., 2013; **Supplementary Table S2**).

These results demonstrate that PGPR can decrease the detrimental effects of toxins by modifying the expression of plant genes encoding for stress response and by directly protecting the plant against the damage produced by pollutants.

#### 4.2.7 Biotic stress

Biotic stress has been reported to induce changes in the photosynthetic activity. Although not all PGPR are effective in biocontrol, they can improve overall plant fitness, inducing a better pathogen response.

Among the considered research articles, leaf gas exchange was measured only by Backes et al. (2021), while fluorescence was taken into account in other studies evaluating the plant response to pathogens in the presence of PGPR. *Burkholderia* strain B25, a PGPR with antifungal activity, was reported to increase the maximum quantum yield of photosystem II in inoculated non-stressed *Hordeum vulgare*, with no enhancement of the CO_2_ assimilation rate, compared to non-inoculated control (**Supplementary Table S2**); however, when B25 was applied to *Hordeum vulgare* infected with *Drechslera teres*, the observed enhancement of F_v_/F_m_ was coupled to an increase in the net CO_2_ assimilation rate compared to non-inoculated stressed plants (**Supplementary Table S2**). Similar results were reported by García-Cristobal et al. (2015), who found pathogenesis-related proteins and ROS detoxification activity increasing in *Oryza sativa* after inoculation with strains *Bacillus* sp. L81 and *Aeromonas* sp. AMG272, known to be effective against *X. campestris*; the protection mechanisms prevented the reduction of the maximum quantum yield of photosystem II instead observed in non-inoculated plants (**Supplementary Table S2**). The quantum yield of PSII in the light was also reported not to change in *G. max* inoculated with *Pseudomonas fluorescens* N21.4, *Stenotrophomonas maltophilia* N5.18, *Chryseobacterium balustinum* Aur9 and infected by *Xanthomonas axonopodis* (Algar et al., 2014; **Supplementary Table S2**). The Authors reported a reduced relative disease index (probably due to a higher isoflavone content observed in plants inoculated with three of the four PGPR used in this research; **Supplementary Table S2**). The enhancement of quantum yield of photosystem II due to the inoculation with PGP bacteria can also have different effects, whether the microorganisms are applied in stressed or unstressed plants. In pot experiments carried out in a growth chamber, Barriuso et al. (2008) analyzed the effect of four Gram-positive PGP rhizobacteria isolated from *Pinus* sp. on the biomass production and photosynthetic activity of *Arabidopsis thaliana* grown under biotic (*Pseudomonas syringae* DC3000) and abiotic (saline, NaCl 60 mM) stress. They showed that these strains determined a significant reduction in the disease index after challenging with *P. syringae* DC3000 and, for three of them, of the chlorotic symptoms under saline stress (**Supplementary Table S2**). They also reported inoculating *A. thaliana* with the PGP strain *Streptomyces* sp. I26 determined a significant F_v_/F_m_ ratio increase only under biotic and abiotic stress conditions and no effect on biomass production (measured as fresh weight) under either stress or control conditions (**Supplementary Table S2**). In contrast, inoculation with *Arthrobacter oxidans* BB1, the unique strain able to stimulate biomass production under all the tested conditions, had no significant effect on the photosynthetic activity of unstressed plants but stimulated photosynthesis in *A. thaliana* exposed to biotic or abiotic stress (Barriuso et al., 2008; **Supplementary Table S2**).

Inoculating *A. thaliana* with M84, a strain belonging to *Curtobacterium* (*Actinobacteria* phylum), a genus commonly found in the phyllosphere microbiome (Mulani et al., 2021), the F_v_/F_m_ ratio and fresh weight varied following the same pattern. It increased only in plants exposed to the biotic stress (**Supplementary Table S2**). In contrast, the treatment of *A. thaliana* with *Bacillus* sp. L81 determined a significant increase in the photosynthetic activity under all tested conditions and when plant growth was unchanged (unstressed and biotic stressed plants; **Supplementary Table S2**).

These results indicated that the photosynthetic activity (even when limited to a single parameter such as the F_v_/F_m_ ratio) could be a valuable tool to differentiate among biocontrol agents that are effective in plant pathogen or abiotic stress protection and have PGP activity only on stressed plants.

On the other hand, the quantum yield of photosystem II has also been reported to decrease after inoculation of stressed plants with PGPR compared to non-inoculated stressed plants. In *A. thaliana* treated with *Phyllobacterium brassicacearum* STM196, Bresson et al. (2014) reported lower F_v_/F_m_ in plants surviving severe drought compared to non-inoculated plants (**Supplementary Table S2**). The authors described a relationship between F_v_/F_m_ and survival probability: inoculation did not affect the *A. thaliana* mortality threshold. However, it delayed and reduced the mortality rate during soil drying, inducing a higher recovery of perishing plants after rewetting. Therefore, the quantum yield of PSII can be a parameter used to predict the fate of plants when subjected to severe stress but cannot give precise insights into what is happening to the whole photosynthetic process.

### 4.3 Effect of abiotic stress on PGPR

Microorganisms are not always immune to the stress to which plants are subjected. Under the same growth conditions, PGP microorganisms can be differently tolerant to stress, and the tolerance can be strain dependent. For instance, Quiñones et al. (2013) showed that nodulation of *Lupinusalbus* was stopped and chlorophyll and xanthophyll content were lower if the plants were inoculated with a Hg-sensitive *Bradyrhizobium canariense* strain and treated with high concentration of HgCl_2_, compared to plants inoculated with an Hg-tolerant strain. In a count of viable PGPR under drought stress, *Acinetobacter calcoaceticus* X128 exhibited greater tolerance to drought among seven microorganisms (*Brevundimonas* sp. X60, *Pseudomonas* sp. X123, *A. calcoaceticus* X128, *Bacillus cereus* Z77, *Paenibacillus polymyxa* SC2, *Bacillus subtilis* GE1, and *Enterobacter cloacae* T026) isolated from the rhizosphere of soils often subjected to severe and repeated water shortage (Liu et al., 2019b). Microorganisms can also exhibit more or less efficient plant growth-promoting traits when grown under stress conditions (Kumari et al., 2015; Wang et al., 2023). The bacterium *Bacillus* sp. JIZ13 showed lower IAA and siderophore production and reduced phosphate solubilizing and ACC deaminase activity when grown in media at increasing NaCl concentration compared to control (Wang et al., 2023).

Bacterial strains applied to the same plant cultivar have been shown to promote plant growth under stress differentially (**Supplementary Table S2**). Naveed et al. (2014) showed that PsJN was more effective compared to FD17 in increasing assimilation rate, probably due to lower non-stomatal limitations (stomatal conductance, F_v_/F_m_ and chlorophyll content were similar). Moreover, Akhtar et al. (2021) demonstrated that growth-promoting activity can be affected by the stress microorganisms are supposed to alleviate in plants. In this exhaustive study, phytohormone production measured in different PGPR was differently affected by drought: IAA and cytokinins produced by *Bacillus* sp., *Azospirillum lipoferum*, *A. brasilense* and a consortium of the three strains significantly decreased under 10% PEG in culture media, while ABA increased significantly in all strains. Moreover, under both control and 10% PEG treatment, IAA, ABA, and cytokinins were produced in higher amounts by the consortium of the three strains rather than the single ones. Ahmed et al. (2021) reported drought-tolerant strain *Enterobacter* sp./*Leclercia adecarboxylata* (MT672579.1) to exhibit higher *in vitro* production of IAA, ACC deaminase, salicylic acid, and the phenolic compound 2, 3-dihydroxybenzoic acid, and phosphate-solubilizing activity at increasing PEG concentrations. The persistence of the inoculated strains might not be the same, independent of the application of the stress. Thus, strains can differ in their colonization efficiency: PsJN CFU/g dry biomass was higher than FD17. This result may explain the higher growth promotion measured in plants treated with PsJN compared to FD17. Kumari et al. (2015) report bacterial colonization of *Pseudomonas* sp. strain AK-1 and *Bacillus* sp. strain SJ-5 to differ regarding CFU/g root in both control and saline conditions. Notably, under control conditions, higher colonization was found in SJ-5 (10^5^ CFU/g root) compared to AK-1 (10^3^ CFU/g root). Under saline conditions, SJ-5 showed better colonization (10^8^ CFU/g root) than AK-1 (10^1^ CFU/g root). It is worth noting that both strains showed an *in vitro* comparable growth under different salt concentrations, highlighting that factors other than the stress treatment can affect the colonization efficiency; the same bacterial strain applied to different cultivars of the same plant species can differently affect plant growth under stress, even if the cultivars’ photosynthetic activity is comparable under control conditions. Barriuso et al. (2008) also showed that bacterial strains selected from *Pinus* sp. that had shown PGPR traits *in vitro* could not stimulate growth in *A. thaliana* grown under optimal conditions.

These observations suggest that, although tolerant to a particular stress, an extensive evaluation of the effectiveness of a plant growth promoter must be done to define eventual changes in the promoting traits. Moreover, microorganisms might have different promoting activities depending on the plant species they apply to.

## 5 Conclusions and future perspectives

This review critically analyzed the literature regarding the effect of PGPR on plant growth and sensing under stressful and non-stressful conditions. Our synthesis revealed non-univocal plant ecophysiological responses to applying PGPR under control and stress conditions.

The analysis of the sole chlorophyll fluorescence does not represent a complete picture of the photosynthetic process. Thus, even if its measure is straightforward, it does not give insights into the mechanisms that drive the photosynthetic and growth processes in the presence of beneficial microorganisms. Fluorometric measurements must, therefore, be coupled to leaf gas exchange and plant anatomical and architectural analysis if more complete information is needed.

Some microorganisms have been shown to stimulate the photosynthetic assimilation rate and efficiency of photosystem II, promoting plant growth. In contrast, others did not produce a positive effect on photosynthesis but did stimulate plant growth by prolonging the vegetative period or increasing leaf number, leaf area and biomass, and root length and biomass.

In most papers where a reduction of the photosynthetic rate was observed, it resulted from a lower stomatal conductance. The latter was caused by an increase in the leaf sucrose content, which might have been triggered by changes in auxin production, transport, and homeostasis induced by the PGPR inoculation. Moreover, if leaf sucrose concentration increases due to a reduced phloem transport, hexokinase can also provoke stomata to close. Non-stomatal limitations to photosynthesis might also occur, mainly due to an increase in sucrose content, which has been proven to reduce RuBisCO content and carboxylative activity. Remarkably, the activity of RuBisCO, the most important enzyme in the Carbon Benson cycle, has been analyzed only in a reduced number of the considered articles, suggesting that we are far from understanding the effect of PGPR on the whole photosynthetic process. Deeper analyses are therefore needed to achieve this objective. Under stress conditions, the increased resistance to desiccation or ion toxicity was achieved by producing primary and secondary metabolites: proline and sugars were used as osmolytes by plants grown under saline conditions and drought.

In contrast, a concomitant increase in enzymes having antioxidant activity reduced ROS concentration in leaves. Different ion absorption and translocation differences were also observed at a root level due to PGPR inoculation. Microorganisms can induce selective absorption by changing root architecture and modifying root hydraulic properties. Although PGPR can enhance plant development and growth under stress conditions, the plant often does not reach the photosynthetic efficiency of non-inoculated plants grown under optimal cultivation conditions.

Plant responses to stress can have different timescales. Sometimes, changes in assimilation rate could only be detected during the late vegetative period, but not at the experiment’s beginning or end. This topic raises a time-connected question. Therefore, it is important to define the best timing to monitor detectable plant changes to reduce time-consuming measurements and increase laboratory efficiency. Depending on the setup and the objectives of the research (testing plant growth-promoting activity under optimal cultivation conditions, analyzing the effect of PGPR compared to non-inoculated plants grown under stressful conditions, or exploring the efficiency of PGPR-induced tolerance in the presence of stress compared to non-inoculated plants grown under optimal conditions), the timescale of the experiment can vary. Short-term (within days) leaf gas exchange measurements can be misleading because changes due to the adaptation to PGPR inoculation can obscure the potential initial benefit of the microorganism. Medium-term experiments seem to fit better when comparing inoculated to uninoculated plants grown in the presence of a specific stress, particularly if a comparison of the efficiency of different single-applied PGPR is the aim of the research. Long-term experiments are recommended when photosynthetic and growth performances of inoculated plants are analyzed compared to control, both in the presence or absence of stress. Moreover, in sequential measurements, the efficiency of PGPR in alleviating the effect of a specific stress must be evaluated in long-term experiments, considering that differences between controls and inoculated plants in the first stages of the application of the stress might probably be undetectable. As growth determines an increase in the time of plant dimension, more extended experiments also need more space; as an increase in competition for light and a possible overlap of canopies can occur, the spatial scale of the investigation must be adequately set.

It is worth considering that the choice of microorganisms with plant growth-promoting traits and their concentration must be carefully considered. Testing *in vitro* growth-promoting traits is not sufficient: microorganisms can show different efficiency in a specific trait; however, the effectiveness of their abilities also depends on their capacity to survive and colonize the soil, rhizo- or the phyllosphere. The production of some metabolites having promoting activity can be modified by the stress that the microorganism is supposed to alleviate in plants. Moreover, the inoculum concentration must also be studied in application tests before the beginning of the experiment, as high concentrations might trigger a negative response in plants, as demonstrated in the two research papers on *Vitis vinifera* considered in this review.

In this work, some articles did not investigate the effect of PGPR on plant biomass or architecture. In some others, only the fresh weight was considered, although water content can strongly affect this parameter. In many experiments, no variations in plant growth were observed in response to PGPR inoculation in both control and stress conditions. If no stimulation in plant biomass, size, or volume occurs compared to non-inoculated plants, can we still call the microorganism “plant growth-promoter”? Can we design experiments on PGPR without considering plant biomass, size or volume? We believe it is essential to integrate plant architecture and leaf physiological measures to test the real potentiality of presumptive PGPR.

## Author contributions

RAJ: Conceptualization, Data curation, Formal analysis, Investigation, Methodology, Visualization, Writing – original draft, Writing – review & editing. FL: Formal analysis, Investigation, Writing – review & editing. AF: Formal analysis, Investigation, Writing – review & editing. MR: Conceptualization, Formal analysis, Funding acquisition, Investigation, Methodology, Supervision, Writing – review & editing.
