# Supplementary material for: A plant’s perception of growth-promoting bacteria and their metabolites
Source: Front Plant Sci. 2024 Jan 24;14:1332864. doi: 10.3389/fpls.2023.1332864 (PMC10848262; doi:10.3389/fpls.2023.1332864)
Supplement: Supplementary file 1 [file DataSheet_1.pdf]

*Supplementary Material*

# A plant's perception of growth-promoting bacteria and their metabolites

**Renée Abou Jaoudé, Francesca Luziatelli, Anna Grazia Ficca, Maurizio Ruzzi***

*** Correspondence:**

Maurizio Ruzzi
ruzzi@unitus.it

**Supplementary Table 1S** Effects of PGPR inoculation on photosynthetic parameters, anatomical traits, nutrients' content, and metabolites measured in plants grown under stress conditions, compared to non-inoculated plants (↑: increase; ↓: decrease; = non-significant variations)

| Plant species | Plant Growth-Promoting Strain | Parameter | PGPR vs. Control | Reference |
|---|---|---|---|---|
| *Arabidopsis thaliana* | *Staphylococcus* sp. I26[a,k,m]<br>*Bacillus* sp. L81[i,m]<br>*Curtobacterium* sp. M84[k]<br>*Arthrobacter oxydans* BB1[h,m] | $F_v/F_m$ | I26 = | Barriuso et al., 2008 |
| | | | L81 ↑ | |
| | | | M84 = | |
| | | | BB1 = | |
| | | Fresh weight | I26 = | |
| | | | L81 = | |
| | | | M84 = | |
| | | | BB1 ↑ | |
| *Arabidopsis thaliana* | *Bacillus subtilis* GB03[g,n] | $F_v/F_m$ | ↑ | Zhang et al., 2008 |
| | | $\Phi_{PSII}$ | ↑ | |
| | | NPQ | ↓ | |
| | | Chlorophyll a/b | = | |
| | | Total chlorophyll | ↑ | |
| | | Leaf hexoses | ↑ | |
| | | ABA in roots | = | |
| | | ABA in shoots | ↓ | |
| | | Sugar sensing | ↓ | |
| *Arabidopsis thaliana* | *Bacillus subtilis* GB03[g,n] | Dry weight | ↑ | Xie et al., 2009 |
| | | Rosette number | ↑ | |
| | | No. silique per plant | ↑ | |
| | | Total chlorophyll | ↑ | |
| | | VOC emission | ↑ | |
| | | Fe | ↑ | |
| *Arabidopsis thaliana* | *Phyllobacterium brassicacearum* STM196[r] | Shoot dry weight | = | Bresson et al., 2013 |
| | | Root dry weight | ↑ | |
| | | Leaf number | ↑ | |
| | | Leaf area | ↑ | |
| | | ABA in shoots | ↑ | |
| | | $CO_2$ assimilation rates | ↓ | |
| | | Leaf sucrose | ↑ | |
| | | Night transpiration rates | ↓ | |
| | | Transpiration rates | ↓ | |
| | | Water use efficiency | = | |
| *Glycine max* | *Pseudomonas fluorescens* N21.4[d,m]<br>*Stenotrophomonas maltophilia* N5.18[d,m]<br>*Chryseobacterium balustinum* Aur9[h]<br>*Curtobacterium* sp. M84[k] | Total fresh weight | N21.4 = | Algar et al., 2014 |
| | | | N51.8 = | |
| | | | Aur9 = | |
| | | | M84 ↓ | |
| | | $\Phi_{PSII}$ | N21.4 ↓ | |
| | | | N51.8 ↓ | |
| | | | Aur9 ↓ | |
| | | | M84 = | |
| | | Ethylene | N21.4 ↓ | |
| | | | N51.8 ↓ | |

| | | | | | |
|---|---|---|---|---|---|
| | | | Aur9 | = | |
| | | | M84 | ↓ | |
| | | Isoflavones | N21.4 | ↑ | |
| | | | N51.8 | ↑ | |
| | | | Aur9 | = | |
| | | | M84 | = | |
| *Glycine max* | *Pseudomonas* sp. AK-1[a,f,h,k,m]<br><br>*Bacillus* sp. SJ-5[a,f,h,k,m] | Shoot length | AK-1 | = | Kumari et al., 2015 |
| | | | SJ-5 | = | |
| | | Root length | AK-1 | ↑ | |
| | | | SJ-5 | ↑ | |
| | | No. leaves | AK-1 | = | |
| | | | SJ-5 | = | |
| | | Total fresh weight | AK-1 | = | |
| | | | SJ-5 | = | |
| | | Lateral roots | AK-1 | ↑ | |
| | | | SJ-5 | ↑ | |
| | | Leaf water content | AK-1 | = | |
| | | | SJ-5 | = | |
| | | Total chlorophyll | AK-1 | = | |
| | | | SJ-5 | = | |
| | | Proline in shoots and leaves | AK-1 | = | |
| | | | SJ-5 | = | |
| | | Proline in roots | AK-1 | = | |
| | | | SJ-5 | = | |
| | | Malondialdehyde | AK-1 | = | |
| | | | SJ-5 | = | |
| | | Lipoxygenase | AK-1 | ↑ | |
| | | | SJ-5 | ↑ | |
| | | Peroxydase in shoots | AK-1 | = | |
| | | | SJ-5 | = | |
| | | Peroxydase in roots | AK-1 | = | |
| | | | SJ-5 | = | |
| | | Catalase | AK-1 | = | |
| | | | SJ-5 | = | |
| | | Superoxide dismutase | AK-1 | = | |
| | | | SJ-5 | = | |
| | | Polyphenol oxidase | AK-1 | = | |
| | | | SJ-5 | = | |
| *Trigonella foenum-graecum* | Consortium of<br><br>*Azotobacter chroococcum*<br><br>*Enterobacter asburiae*<br><br>*Lactococcus lactis* | Shoot length | ↑ | | Bisht et al., 2022 |
| | | Root length | = | | |
| | | Shoot dry weight | = | | |
| | | Root dry weight | ↑ | | |
| | | Leaf area | ↑ | | |
| | | No. leaves | = | | |
| | | $CO_2$ assimilation rates | ↑ | | |
| | | Stomatal conductance | ↑ | | |
| | | Transpiration rates | ↑ | | |
| | | Intercellular $CO_2$ conc. | ↑ | | |
| | | Carotenoids | ↑ | | |
| | | Chlorophyll a | ↑ | | |

| | | | | |
|---|---|---|---|---|
| | | Chlorophyll b | = | |
| | | Total chlorophyll | ↑ | |
| | | N | ↑ | |
| | | Proteins | ↑ | |
| *Cicer arietinum* | *Mesorhizobium ciceri* (MC)[a,f,j, k,m]<br>*Serratia marcescens* SF3[a,f,k,m]<br>*Serratia* sp. ST9[a,f,k,m] | Total chlorophyll | MC = | Shahzad et al., 2014 |
| | | | SF3 = | |
| | | | ST9 = | |
| | | | MC + SF3 ↑ | |
| | | | MC + ST9 ↑ | |
| | | $CO_2$ assimilation rates | MC = | |
| | | | SF3 = | |
| | | | ST9 = | |
| | | | MC + SF3 ↑ | |
| | | | MC + ST9 ↑ | |
| | | Transpiration rates | MC = | |
| | | | SF3 ↑ | |
| | | | ST9 ↑ | |
| | | | MC + SF3 ↑ | |
| | | | MC + ST9 ↑ | |
| | | Plant height | MC ↑ | |
| | | | SF3 ↑ | |
| | | | ST9 ↑ | |
| | | | MC + SF3 ↑ | |
| | | | MC + ST9 ↑ | |
| | | Shoot dry weight | MC ↑ | |
| | | | SF3 ↑ | |
| | | | ST9 ↑ | |
| | | | MC + SF3 ↑ | |
| | | | MC + ST9 ↑ | |
| | | Grain yield | MC ↑ | |
| | | | SF3 ↑ | |
| | | | ST9 ↑ | |
| | | | MC + SF3 ↑ | |
| | | | MC + ST9 ↑ | |
| | | No. Pods | MC ↑ | |
| | | | SF3 ↑ | |
| | | | ST9 ↑ | |
| | | | MC + SF3 ↑ | |
| | | | MC + ST9 ↑ | |
| | | Root length | MC ↑ | |
| | | | SF3 ↑ | |
| | | | ST9 ↑ | |
| | | | MC + SF3 ↑ | |
| | | | MC + ST9 ↑ | |
| | | Root dry weight | MC ↑ | |
| | | | SF3 ↑ | |
| | | | ST9 ↑ | |
| | | | MC + SF3 ↑ | |
| | | | MC + ST9 ↑ | |
| | | No. Nodules | MC ↑ | |

| | | | | | |
|---|---|---|---|---|---|
| | | | SF3 | ↑ | |
| | | | ST9 | ↑ | |
| | | | MC + SF3 | ↑ | |
| | | | MC + ST9 | ↑ | |
| | | Nodule dry weight | MC | ↑ | |
| | | | SF3 | ↑ | |
| | | | ST9 | ↑ | |
| | | | MC + SF3 | ↑ | |
| | | | MC + ST9 | ↑ | |
| | | Protein in grains | MC | = | |
| | | | SF3 | = | |
| | | | ST9 | = | |
| | | | MC + SF3 | ↑ | |
| | | | MC + ST9 | ↑ | |
| *Phaseolus coccineus* | *Bacillus pumilus* S4[k,m]<br><br>*Bacillus mycoides* S7[h] | $CO_2$ assimilation rates | S4 | = | Stefan et al., 2013 |
| | | | S7 (day 28-42) | ↑ | |
| | | | S4+S7 (day 28-42) | ↑ | |
| | | Transpiration rates | S4 | = | |
| | | | S7 | = | |
| | | | S4+S7 (day 28) | ↑ | |
| | | Water use efficiency | S4 | = | |
| | | | S7 (day 28-96) | ↑ | |
| | | | S4+S7 (day 28-42) | ↑ | |
| | | Total chlorophyll | S4 (day 28) | ↑ | |
| | | | S7 | = | |
| | | | S4 + S7 | = | |
| | | Grain yield | S4 | = | |
| | | | S7 | ↑ | |
| | | | S4+S7 | ↑ | |
| *Panicum virgatum* | *Burkholderia phytofirmans* PsJN[a,h] | $CO_2$ assimilation rates | ↑ | | Wang et al., 2015 |
| | | Stomatal conductance | ↑ | | |
| | | Water use efficiency | ↑ | | |
| | | $C_i$/Ca | ↓ | | |
| | | Shoot dry weight | ↑ | | |
| | | Root dry weight | ↑ | | |
| | | Total dry weight | ↑ | | |
| | | Leaf area | ↑ | | |
| | | Height | ↑ | | |
| | | Root length | ↑ | | |
| | | Root surface area | ↑ | | |
| | | Root average diameter | ↑ | | |
| *Triticum aestivum* | Consortium of<br><br>*Bacillus* sp.[e,h,p,]<br>*Azospirillum lipoferum*[e,h,p,q]<br>*Azospirillum brasilense*[e,h,p,q] | $CO_2$ assimilation rates | ↑ | | Akhtar et al., 2021 |
| | | Transpiration rates | ↑ | | |
| | | Stomatal conductance | ↑ | | |
| | | Proline | ↑ | | |
| | | Sugars | = | | |
| | | Proteins | ↑ | | |
| | | Relative water content | = | | |
| | | Chlorophyll a | ↑ | | |
| | | Chlorophyll b | ↑ | | |

| | | | | |
|---|---|---|---|---|
| | | Carotenoids | ↑ | |
| | | Peroxidase | ↑ | |
| | | Catalase | = | |
| | | Superoxide dismutase | = | |
| | | N in roots | = | |
| | | P in roots | = | |
| | | K in roots | ↑ | |
| | | N in shoots | ↑ | |
| | | P in shoots | = | |
| | | K in shoots | ↑ | |
| | | N in grains | ↑ | |
| | | P in grains | ↑ | |
| | | K in grains | ↑ | |
| | | Total dry weight | = | |
| | | Yield | ↑ | |
| | | IAA in grains | ↑ | |
| | | Cytokinins - grains | ↑ | |
| | | ABA in grains | ↑ | |
| | | Electrolyte leakage | = | |
| *Zea mays* | *Pseudomonas fluorescens* Aur6[h,m] | $F_0$ | = | Grijalbo et al., 2013 |
| | | $F_v/F_m$ | = | |
| | | Hill reaction | ↑ | |
| | | Chlorophyll a/b | ↑ | |
| | | Total Chlorophyll | ↑ | |
| *Zea mays* cv Marzuka<br><br>*Zea mays* cv Kaleo | *Burkholderia phytofirmans* PsJN[a,h]<br><br>*Enterobacter* sp. FD17[a,b,c,k] | $CO_2$ assimilation rates | PsJN both cv ↑ | Naveed et al., 2014 |
| | | | FD17 both cv ↑ | |
| | | Stomatal conductance | PsJN both cv ↑ | |
| | | | FD17 both cv = | |
| | | Transpiration rates | PsJN both cv ↑ | |
| | | | FD17 both cv ↑ | |
| | | Vapour pressure deficit | PsJN both cv = | |
| | | | FD17 both cv = | |
| | | Relative water content | PsJN both cv ↑ | |
| | | | FD17 both cv ↑ | |
| | | $F_v/F_m$ | PsJN both cv ↑ | |
| | | | FD17 both cv = | |
| | | Membrane permeability | PsJN both cv ↓ | |
| | | | FD17 both cv = | |
| | | Total chlorophyll | PsJN both cv ↑ | |
| | | | FD17 both cv ↑ | |
| | | No. Leaves | PsJN both cv ↑ | |
| | | | FD17 both cv = | |
| | | Leaf area | PsJN both cv ↑ | |
| | | | FD17 both cv ↑ | |
| | | Shoot dry weight | PsJN both cv ↑ | |
| | | | FD17 both cv ↑ | |
| | | Root dry weight | PsJN both cv ↑ | |
| | | | FD17 both cv ↑ | |
| *Zea mays* | *Bacillus megaterium*[s] | Electrolyte leakage | = | |
| | | Shoot dry weight | = | |

| | | | | |
|---|---|---|---|---|
| | | Root dry weight | ↓ | |
| | | Shoot water content | ↑ | |
| | | Stomatal conductance | = | |
| | | $\Phi_{PSII}$ | = | |
| | | $CO_2$ assimilation rates | = | |
| | | Water use efficiency | = | |
| | | Osmotic hydraulic conductivity | ↑ | Romero-Munar and Aroca, 2023 |
| | | Hydrostatic root hydraulic conductivity | = | |
| | | ABA in sap | = | |
| | | Jasmonic acid in sap | = | |
| | | IAA in sap | = | |
| | | Salycilic acid in sap | = | |
| | | Ja-Ile in sap | ↑ | |
| | | Acquaporins | ↓ | |
| *Solanum lycorpersicum* parental line (cv. Pearson)<br><br>*Solanum lycorpersicum* mutant line "*never ripe" (ethylene-insensitive)* | *Bacillus megaterium*[s]<br><br>*Enterobacter* C7 | Total fresh weight | Bm in wt ↑<br>C7 in wt ↑<br>Bm in nr =<br>C7 in nr ↑ | Ibort et al., 2018 |
| | | Root fresh weigth | Bm in wt =<br>C7 in wt =<br>Bm in nr =<br>C7 in nr = | |
| | | Shoot fresh weight | Bm in wt ↑<br>C7 in wt ↑<br>Bm in nr ↑<br>C7 in nr ↑ | |
| | | Root P | Bm in wt =<br>C7 in wt ↑<br>Bm in nr ↑<br>C7 in nr = | |
| | | P-transporter 1 | Bm in wt =<br>C7 in wt =<br>Bm in nr ↑<br>C7 in nr = | |
| | | P-transporter 2 | Bm in wt =<br>C7 in wt =<br>Bm in nr =<br>C7 in nr ↑ | |
| | | Ascorbate peroxidase | Bm in wt ↓<br>C7 in wt ↓<br>Bm in nr =<br>C7 in nr = | |
| | | Reduced glutathione | Bm in wt =<br>C7 in wt ↓<br>Bm in nr =<br>C7 in nr = | |

| | | | | | |
|---|---|---|---|---|---|
| | | Oxidized glutathione | Bm in wt | ↓ | |
| | | | C7 in wt | ↓ | |
| | | | Bm in nr | = | |
| | | | C7 in nr | = | |
| | | Ascorbic acid | Bm in wt | ↑ | |
| | | | C7 in wt | ↓ | |
| | | | Bm in nr | ↓ | |
| | | | C7 in nr | ↓ | |
| | | Total glutathione | Bm in wt | ↑ | |
| | | | C7 in wt | ↓ | |
| | | | Bm in nr | ↓ | |
| | | | C7 in nr | ↓ | |
| *Sambucus williamsii* | *Acinetobacter calcoaceticus* X128[e,h,q] | $CO_2$ assimilation rates | ↑ | | Liu et al., 2019b |
| | | Stomatal conductance | ↑ | | |
| | | Intercellular $CO_2$ conc. | = | | |
| | | Relative water content | = | | |
| | | Cytokinins in roots | = | | |
| | | Cytokinins in shoots | ↑ | | |
| | | ABA in roots | ↑ | | |
| | | ABA in shoots | = | | |
| | | Relative conductivity | = | | |
| | | Shoot dry weight | ↑ | | |
| | | Root dry weight | ↑ | | |
| *Trema micrantha*<br><br>*Cariniana estrellensis* | *Azospirillum brasilense* Ab-V5[j]<br><br>*Bacillus* sp. (BA)[h]<br><br>*Azomonas* sp. (AM)<br><br>*Azorhizophillus* sp. (AR) | Water potential | Ab-V5 in *T.m.* | = | Nunes Tiepo et al., 2018 |
| | | | Ab-V5 in *C.e.* | = | |
| | | | BA in *T.m.* | = | |
| | | | BA in *C.e.* | = | |
| | | | AM in T.m. | = | |
| | | | AM in *C.e.* | = | |
| | | | AR in *T.m.* | ↓ | |
| | | | AR in *C.e.* | = | |
| | | Proline | Ab-V5 in *T.m.* | = | |
| | | | Ab-V5 in *C.e.* | = | |
| | | | BA in *T.m.* | = | |
| | | | BA in *C.e.* | = | |
| | | | AM in T.m. | ↓ | |
| | | | AM in *C.e.* | = | |
| | | | AR in *T.m.* | = | |
| | | | AR in *C.e.* | = | |
| | | Thiobarbituric acid reactive substances | Ab-V5 in *T.m.* | = | |
| | | | Ab-V5 in *C.e.* | = | |
| | | | BA in *T.m.* | = | |
| | | | BA in *C.e.* | = | |
| | | | AM in T.m. | = | |
| | | | AM in *C.e.* | = | |
| | | | AR in *T.m.* | = | |
| | | | AR in *C.e.* | = | |
| | | $H_2O_2$ | Ab-V5 in *T.m.* | = | |
| | | | Ab-V5 in *C.e.* | = | |

| | | | | | |
|---|---|---|---|---|---|
| | | | BA in *T.m.* | ↓ | |
| | | | BA in *C.e.* | ↓ | |
| | | | AM in T.m. | = | |
| | | | AM in *C.e.* | = | |
| | | | AR in *T.m.* | = | |
| | | | AR in *C.e.* | ↑ | |
| | | Root dry weight | Ab-V5 in *T.m.* | = | |
| | | | Ab-V5 in *C.e.* | = | |
| | | | BA in *T.m.* | = | |
| | | | BA in *C.e.* | = | |
| | | | AM in T.m. | = | |
| | | | AM in *C.e.* | = | |
| | | | AR in *T.m.* | = | |
| | | | AR in *C.e.* | = | |
| | | Shoot dry weight | Ab-V5 in *T.m.* | = | |
| | | | Ab-V5 in *C.e.* | = | |
| | | | BA in *T.m.* | = | |
| | | | BA in *C.e.* | = | |
| | | | AM in T.m. | = | |
| | | | AM in *C.e.* | = | |
| | | | AR in *T.m.* | = | |
| | | | AR in *C.e.* | = | |
| | | Root:shoot ratio | Ab-V5 in *T.m.* | = | |
| | | | Ab-V5 in *C.e.* | = | |
| | | | BA in *T.m.* | = | |
| | | | BA in *C.e.* | ↑ | |
| | | | AM in T.m. | = | |
| | | | AM in *C.e.* | ↑ | |
| | | | AR in *T.m.* | = | |
| | | | AR in *C.e.* | = | |
| | | $CO_2$ assimilation rates | Ab-V5 in *T.m.* | = | |
| | | | Ab-V5 in *C.e.* | = | |
| | | | BA in *T.m.* | = | |
| | | | BA in *C.e.* | = | |
| | | | AM in T.m. | = | |
| | | | AM in *C.e.* | = | |
| | | | AR in *T.m.* | = | |
| | | | AR in *C.e.* | ↓ | |
| | | Stomatal conductance | Ab-V5 in *T.m.* | = | |
| | | | Ab-V5 in *C.e.* | = | |
| | | | BA in *T.m.* | = | |
| | | | BA in *C.e.* | = | |
| | | | AM in T.m. | = | |
| | | | AM in *C.e.* | = | |
| | | | AR in *T.m.* | = | |
| | | | AR in *C.e.* | ↓ | |
| | | Intercellular $CO_2$ concentration | Ab-V5 in *T.m.* | = | |
| | | | Ab-V5 in *C.e.* | = | |
| | | | BA in *T.m.* | = | |
| | | | BA in *C.e.* | = | |

| | | | | | |
|---|---|---|---|---|---|
| | | | AM in T.m. | = | |
| | | | AM in *C.e.* | = | |
| | | | AR in *T.m.* | = | |
| | | | AR in *C.e.* | = | |
| | | Carboxylation efficiency | Ab-V5 in *T.m.* | = | |
| | | | Ab-V5 in *C.e.* | = | |
| | | | BA in *T.m.* | = | |
| | | | BA in *C.e.* | = | |
| | | | AM in T.m. | = | |
| | | | AM in *C.e.* | = | |
| | | | AR in *T.m.* | = | |
| | | | AR in *C.e.* | = | |
| | | Total soluble sugars in leaves | Ab-V5 in *T.m.* | = | |
| | | | Ab-V5 in *C.e.* | = | |
| | | | BA in *T.m.* | = | |
| | | | BA in *C.e.* | ↓ | |
| | | | AM in T.m. | = | |
| | | | AM in *C.e.* | = | |
| | | | AR in *T.m.* | = | |
| | | | AR in *C.e.* | ↓ | |
| | | Total soluble sugars in roots | Ab-V5 in *T.m.* | ↓ | |
| | | | Ab-V5 in *C.e.* | = | |
| | | | BA in *T.m.* | = | |
| | | | BA in *C.e.* | = | |
| | | | AM in T.m. | ↓ | |
| | | | AM in *C.e.* | = | |
| | | | AR in *T.m.* | = | |
| | | | AR in *C.e.* | = | |
| | | Starch in leaves | Ab-V5 in *T.m.* | = | |
| | | | Ab-V5 in *C.e.* | ↑ | |
| | | | BA in *T.m.* | = | |
| | | | BA in *C.e.* | = | |
| | | | AM in T.m. | = | |
| | | | AM in *C.e.* | = | |
| | | | AR in *T.m.* | = | |
| | | | AR in *C.e.* | = | |
| | | Starch in roots | Ab-V5 in *T.m.* | ↓ | |
| | | | Ab-V5 in *C.e.* | ↑ | |
| | | | BA in *T.m.* | ↓ | |
| | | | BA in *C.e.* | = | |
| | | | AM in T.m. | = | |
| | | | AM in *C.e.* | ↑ | |
| | | | AR in *T.m.* | = | |
| | | | AR in *C.e.* | = | |
| | | Protein in leaves | Ab-V5 in *T.m.* | = | |
| | | | Ab-V5 in *C.e.* | ↑ | |
| | | | BA in *T.m.* | = | |
| | | | BA in *C.e.* | = | |
| | | | AM in T.m. | = | |
| | | | AM in *C.e.* | = | |

| | | | | |
|---|---|---|---|---|
| | | AR in *T.m.* | = | |
| | | AR in *C.e.* | ↑ | |
| *Vitis vinifera* | *Burkholderia phytofirmans* PsJN[a,h] | $CO_2$ assimilation rates | ↑ | Ait Barka et al., 2006 |
| | | Oxygen production | ↑ | |
| | | Total dry weight | ↑ | |
| | | Shoot dry weight | ↑ | |
| | | Root dry weight | ↑ | |
| | | Electrolytes leakage | = | |
| | | Proline in shoots | ↑ | |
| | | Proline in leaves | ↑ | |
| | | Proline in roots | ↑ | |
| | | Starch in shoots | ↑ | |
| | | Starch in leaves | ↑ | |
| | | Starch in roots | = | |
| | | Phenols | ↑ | |
| *Vitis vinifera* | *Burkholderia phytofirmans* PsJN[a,h] | $CO_2$ assimilation rates | ↓ | Fernandez et al., 2012 |
| | | Stomatal conductance | = | |
| | | Intercellular $CO_2$ conc. | = | |
| | | $\Phi_{PSII}$ | = | |
| | | Total chlorophyll | ↓ | |
| | | Carotenoids | ↓ | |
| | | Starch | ↑ | |
| | | Total soluble sugars | ↑ | |
| | | Pyruvate | ↓ | |
| | | Phosphoenolpyrivate | ↓ | |
| *Salicornia ramosissima* | Consortium of<br>*Thalassospira australica* SRT8[a]<br>*Pseudarthrobacter oxydans* SRT15[c,h,j]<br>*Vibrio neocaledonicus* SRT1[c,h,j,k,m] | Relative growth rates | ↑ | Mesa-Marín et al., 2020 |
| | | Stem branches increment | ↑ | |
| | | Water content | = | |
| | | $CO_2$ assimilation rates | = | |
| | | Stomatal conductance | = | |
| | | Intercellular $CO_2$ conc. | = | |
| | | Water use efficiency | = | |
| | | $F_m$ | ↓ | |
| | | $F_v/F_m$ | ↓ | |
| | | Absorbed energy flux | = | |
| | | Trapped energy flux | = | |
| | | ET energy flux* | = | |
| | | Dissipated energy flux | ↑ | |
| | | Ca in leaves | ↑ | |
| | | Ca in roots | = | |
| | | K in leaves | = | |
| | | K in roots | = | |
| | | Mg in leaves | ↑ | |
| | | Mg in roots | = | |
| | | Mn in leaves | ↑ | |
| | | Mn in roots | = | |
| | | Na in leaves | ↑ | |
| | | Na in roots | = | |
| | | P in leaves | = | |
| | | P in roots | = | |

*Electron transport energy flux

[a]ACC deaminase

[b]Biocontrol

[c]Biofilm formation

[d]Chitinases production

[e]Cytokinins production

[f]Exopolysaccharide production

[g]Glucose/ABA sensing

[h]IAA production

[i]Induction of systemic resistance

[j]N fixation

[k]Phosphate solubilization

[l]Phytase production

[m]Siderophore production

[n]VOCs production

[o]Cd tolerance

[p]ABA production

[q]Osmolyte production

[r] Effect on root architechture

[s] Effect on root hydraulic properties

**Supplementary Table 2S.** Effects of PGPR inoculation on photosynthetic parameters, anatomical traits, nutrients' content, and metabolites measured in plants grown under non-stress conditions, compared to non-inoculated plants (↑: increase; ↓: decrease; = non-significant variations)

| Plant species | Plant Growth-Promoting strain | Stress | Parameter | PGPR stress vs. Non-inoculated | | Reference |
|---|---|---|---|---|---|---|
| *Arabidopsis thaliana* | *Staphylococcus* sp. I26[a,k,m]<br>*Bacillus* sp. L81[i,m]<br>*Curtobacterium* sp. M84[k]<br>*Arthrobacter oxydans* BB1[h,m] | Biotic<br>*Pseudomonas syringae* DC3000<br>Abiotic<br>*Salinity (60 mM NaCl)* | $F_v/F_m$ | I26 biotic | ↑ | Barriuso et al., 2008 |
| | | | | I26 abiotic | ↑ | |
| | | | | L81 biotic | ↑ | |
| | | | | L81 abiotic | ↑ | |
| | | | | M84 biotic | ↑ | |
| | | | | M84 abiotic | = | |
| | | | | BB1 biotic | ↑ | |
| | | | | BB1 abiotic | ↑ | |
| | | | Total fresh weight | I26 biotic | = | |
| | | | | I26 abiotic | ↑ | |
| | | | | L81 biotic | = | |
| | | | | L81 abiotic | ↑ | |
| | | | | M84 biotic | ↑ | |
| | | | | M84 abiotic | = | |
| | | | | BB1 biotic | ↑ | |
| | | | | BB1 abiotic | ↑ | |
| | | | Relative disease index | I26 biotic | ↓ | |
| | | | | I26 abiotic | ↓ | |
| | | | | L81 biotic | ↓ | |
| | | | | L81 abiotic | ↓ | |
| | | | | M84 biotic | ↓ | |
| | | | | M84 abiotic | = | |
| | | | | BB1 biotic | ↓ | |
| | | | | BB1 abiotic | ↓ | |
| *Arabidopsis thaliana* | *Bacillius subtilis* GB03[g,n] | Abiotic<br>Fe deficiency | Chlorophyll a/b | ↑ | | Zhang et al., 2009 |
| | | | $F_v/F_m$ | ↑ | | |
| | | | Acid extrusion | ↑ | | |
| | | | $Fe^{3+}$-chelate reductase | ↑ | | |
| | | | $Fe^{2+}$-transported | ↑ | | |
| | | | Fe-deficiency Induced Transcription Factor 1 | ↑ | | |
| | | | Iron | ↑ | | |
| *Arabidopsis thaliana* | *Phyllobacterium brassicacearum* STM196[r] | Drought | Shoot dry weight | ↑ | | Bresson et al., 2013 |
| | | | Root dry weight | ↑ | | |
| | | | Leaf number | ↑ | | |
| | | | Leaf area | ↑ | | |
| | | | ABA in shoots | ↑ | | |
| | | | $CO_2$ assimilation rates | ↓ | | |
| | | | Leaf sucrose | ↑ | | |
| | | | Night transpiration rates | ↓ | | |
| | | | Transpiration rates | ↓ | | |
| | | | Water use efficiency | ↑ | | |
| *Cicer arietinum* | *Mesorhizobium ciceri* (MC)[a,f,k,m]<br>*Serratia marcescens* SF3<br>*Serratia* sp. ST9 | Abiotic<br>*Irrigated vs rainfed farming system P- enrichment* | Total chlorophyll | MC | ↑ | Shahzad et al., 2014 |
| | | | | SF3 | = | |
| | | | | ST9 | = | |
| | | | | MC + SF3 | ↑ | |
| | | | | MC + ST9 | ↑ | |

| | | | | | | |
|---|---|---|---|---|---|---|
| | | | $CO_2$ assimilation rates | MC | = | |
| | | | | SF3 | = | |
| | | | | ST9 | = | |
| | | | | MC + SF3 | ↑ | |
| | | | | MC + ST9 | ↑ | |
| | | | Transpiration rates | MC | = | |
| | | | | SF3 | = | |
| | | | | ST9 | = | |
| | | | | MC + SF3 | ↑ | |
| | | | | MC + ST9 | ↑ | |
| | | | Plant height | MC | ↑ | |
| | | | | SF3 | ↑ | |
| | | | | ST9 | ↑ | |
| | | | | MC + SF3 | ↑ | |
| | | | | MC + ST9 | ↑ | |
| | | | Shoot dry weight | MC | ↑ | |
| | | | | SF3 | ↑ | |
| | | | | ST9 | ↑ | |
| | | | | MC + SF3 | ↑ | |
| | | | | MC + ST9 | ↑ | |
| | | | Grain yield | MC | ↑ | |
| | | | | SF3 | ↑ | |
| | | | | ST9 | ↑ | |
| | | | | MC + SF3 | ↑ | |
| | | | | MC + ST9 | ↑ | |
| | | | No. Pods | MC | ↑ | |
| | | | | SF3 | ↑ | |
| | | | | ST9 | ↑ | |
| | | | | MC + SF3 | ↑ | |
| | | | | MC + ST9 | ↑ | |
| | | | Root length | MC | ↑ | |
| | | | | SF3 | ↑ | |
| | | | | ST9 | ↑ | |
| | | | | MC + SF3 | ↑ | |
| | | | | MC + ST9 | ↑ | |
| | | | Root dry weight | MC | ↑ | |
| | | | | SF3 | ↑ | |
| | | | | ST9 | ↑ | |
| | | | | MC + SF3 | ↑ | |
| | | | | MC + ST9 | ↑ | |
| | | | No. Nodules | MC | ↑ | |
| | | | | SF3 | ↑ | |
| | | | | ST9 | ↑ | |
| | | | | MC + SF3 | ↑ | |
| | | | | MC + ST9 | ↑ | |
| | | | Nodule dry weight | MC | ↑ | |
| | | | | SF3 | ↑ | |
| | | | | ST9 | ↑ | |
| | | | | MC + SF3 | ↑ | |
| | | | | MC + ST9 | ↑ | |
| | | | Protein in grains | MC | ↑ | |
| | | | | SF3 | = | |
| | | | | ST9 | = | |
| | | | | MC + SF3 | ↑ | |

| | | | | | |
|---|---|---|---|---|---|
| | | | | MC + ST9 ↑ | |
| *Fragaria vesca* var. Rociera | Consortium of<br><br>*Pseudomonas* SDT3[k,m]<br>*B. zhangzhouensi* HPJ40[c,j,k,m]<br>*B. velezensis* SMT38[a,c,j,m]<br>*P. oxydans* SRT15[c,h,j,k]<br>*V. paradoxus* S110[a,c,h,j,m] | Abiotic<br>P deficiency | Root and shoot dry weight | ↑ (+ inorganic P) | Valle-Romero et al., 2023 |
| | | | $CO_2$ assimilation rates | ↑ | |
| | | | Stomatal conductance | ↑ | |
| | | | Intercellular $CO_2$ concentration | ↓ | |
| | | | Water use efficiency | ↑ | |
| | | | Mesophyll conductance | ↑ (+ inorganic P) | |
| | | | $V_{cmax}$ | ↑ | |
| | | | $F_v/F_m$ | ↑ | |
| | | | $F_{PSII}$ | ↑ | |
| | | | ETR | ↑ | |
| | | | P | ↑ | |
| | | | K | ↑ | |
| | | | C/N | ↑ | |
| *Glomus versiforme* | *Micrococcus yunnanensis*[k,m] | Abiotic<br>*Salinity* (5, 10, 15 dS $m^{-1}$) | Plant height | ↑ | Afrangan et al., 2023 |
| | | | Chlorophyll a | ↑ | |
| | | | Chlorophyll b | ↑ | |
| | | | Carotenoids | ↑ | |
| | | | Oil content | ↑ | |
| | | | Catalase | ↑ | |
| | | | Polyphenol oxidase | ↑ | |
| | | | Superoxide dismutase | ↑ | |
| | | | $CO_2$ assimilation rates | ↑ | |
| | | | Stomatal conductance | ↑ | |
| | | | Transpiration rates | ↑ | |
| | | | Shoot $Na^+$ | = | |
| | | | Root $Na^+$ | = | |
| | | | Shoot $K^+$ | ↑ 10 and 15 dS $m^{-1}$ | |
| | | | Root $K^+$ | ↑ | |
| | | | Grain yield | ↑ | |
| *Glycine max* | *Pseudomonas fluorescens* N21.4[d,m]<br><br>*Stenotrophomonas maltophilia* N5.18[d,m]<br><br>*Chryseobacterium balustinum* Aur9[h]<br><br>*Curtobacterium* sp. M84[k] | Biotic<br>*Xantomonas axonopodis* pv. *glycines* | Total fresh weight | N21.4 = | Algar et al., 2014 |
| | | | | N51.8 = | |
| | | | | Aur9 = | |
| | | | | M84 = | |
| | | | $\Phi_{PSII}$ | N21.4 = | |
| | | | | N51.8 = | |
| | | | | Aur9 = | |
| | | | | M84 ↓ | |
| | | | Relative disease index | N21.4 ↓ | |
| | | | | N51.8 ↓ | |
| | | | | Aur9 ↓ | |
| | | | | M84 ↓ | |
| | | | Isoflavones | N21.4 ↑ | |
| | | | | N51.8 = | |
| | | | | Aur9 ↑ | |
| | | | | M84 ↑ | |
| *Glycine max* | *Pseudomonas* sp. AK-1[a,f,h,k,m]<br><br>*Bacillus* sp. SJ-5[a,f,h,k,m] | Abiotic<br>*Salinity* | Shoot length | AK-1 ↑ | Kumari et al., 2015 |
| | | | | SJ-5 ↑ | |
| | | | Root length | AK-1 ↑ | |
| | | | | SJ-5 ↑ | |
| | | | No. leaves | AK-1 ↑ | |
| | | | | SJ-5 ↑ | |

| | | | | | | |
|---|---|---|---|---|---|---|
| | | | Total fresh weight | AK-1 | ↑ | |
| | | | | SJ-5 | ↑ | |
| | | | Lateral roots | AK-1 | ↑ | |
| | | | | SJ-5 | ↑ | |
| | | | Leaf water content | AK-1 | ↑ | |
| | | | | SJ-5 | ↑ | |
| | | | Total chlorophyll | AK-1 | ↑ | |
| | | | | SJ-5 | ↑ | |
| | | | Proline in shoots and leaves | AK-1 | ↓ | |
| | | | | SJ-5 | ↓ | |
| | | | Proline in roots | AK-1 | ↑ | |
| | | | | SJ-5 | ↑ | |
| | | | Malondialdehyde | AK-1 | ↓ | |
| | | | | SJ-5 | ↓ | |
| | | | Lipoxygenase | AK-1 | ↑ | |
| | | | | SJ-5 | ↑ | |
| | | | Peroxydase in shoots | AK-1 | ↓ | |
| | | | | SJ-5 | ↓ | |
| | | | Peroxydase in roots | AK-1 | ↑ | |
| | | | | SJ-5 | ↑ | |
| | | | Catalase | AK-1 | ↓ | |
| | | | | SJ-5 | ↓ | |
| | | | Superoxide dismutase | AK-1 | ↓ | |
| | | | | SJ-5 | ↓ | |
| | | | Polyphenol oxidase | AK-1 | ↓ | |
| | | | | SJ-5 | ↓ | |
| *Hordeum vulgare* | *Burkholderia* sp. *B25*[b] | Biotic *Drechslera teres* | Y(NA) | | = | Backes et al., 2021 |
| | | | Y(ND) | | = | |
| | | | Y(I) | | ↑ | |
| | | | Y(II) | | = | |
| | | | NPQ | | = | |
| | | | NO | | ↑ | |
| | | | $Y_{CEF}$ | | = | |
| | | | $F_v/F_m$ | | ↑ | |
| | | | ETRI | | = | |
| | | | ETRII | | = | |
| | | | $CO_2$ assimilation rates | | ↑ | |
| | | | Intercellular $CO_2$ concentration | | = | |
| | | | Transpiration rates | | = | |
| | | | Stomatal conductance | | = | |
| | | | Dark transpiration rates | | = | |
| | | | Dark stomatal conductance | | = | |
| | | | Dark respiration | | = | |
| *Lolium multiflorum*<br><br>*Glycine max* | *Bradyrhizobium* sp. YL-6[a,h,k,m,o] | Abiotic *Heavy metals (Cd)* | Shoot dry weight | *L.m.* | ↑ | Guo and Chi, 2014 |
| | | | | *G. max* | = | |
| | | | Root dry weight | *L.m.* | = | |
| | | | | *G. max* | = | |
| | | | Chlorophyll a | *L.m.* | ↑ | |
| | | | | *G. max* | ↑ | |
| | | | Chlorophyll b | *L.m.* | ↑ | |
| | | | | *G. max* | ↑ | |
| | | | Carotenoids | *L.m.* | ↑ | |
| | | | | *G. max* | ↑ | |
| | | | Chlorophyll a/b | *L.m.* | ↑ | |

| | | | | | |
|---|---|---|---|---|---|
| | | | | *G. max* ↑ | |
| | | | Mg in leaves | *L.m.* ↑ | |
| | | | | *G. max* ↓ | |
| | | | Fe in leaves | *L.m.* = | |
| | | | | *G. max* ↑ | |
| | | | Cd in leaves | *L.m.* = | |
| | | | | *G. max* ↓ | |
| | | | Cd in roots | *L.m.* ↑ | |
| | | | | *G. max* ↓ | |
| | | | Cd in shoots | *L.m.* = | |
| | | | | *G. max* ↓ | |
| | | | Root Cd uptake | *L.m.* ↑ | |
| | | | | *G. max* ↓ | |
| | | | Shoot Cd uptake | *L.m.* ↑ | |
| | | | | *G. max* = | |
| *Lolium multiflorum* | *Burkholderia* sp. D54 | Abiotic<br>*Heavy metals (Cd, Zn, As, Pb)* | Chlorophyll a | = | Guo et al., 2014 |
| | | | Chlorophyll b | ↑ | |
| | | | Carotenoids | ↑ | |
| | | | $CO_2$ assimilation rates | = | |
| | | | Intercellular $CO_2$ concentration | = | |
| | | | Transpiration rates | = | |
| | | | Water use efficiency | = | |
| | | | Root dry weight | ↑ | |
| | | | Shoot dry weight | ↑ | |
| | | | Zn in roots | = | |
| | | | Zn in shoots | ↓ | |
| | | | Cd in roots | = | |
| | | | Cd in shoots | ↓ | |
| | | | As in roots | ↑ | |
| | | | As in shoots | = | |
| | | | Pb in roots | ↑ | |
| | | | Pb in shoots | ↑ | |
| | | | Transfer factor | Zn, As, Cd ↓ | |
| | | | | Pb = | |
| | | | Bioaccumulation factor | Zn, As = | |
| | | | | Cd ↓ | |
| | | | | Pb ↑ | |
| *Oryza sativa* | *Bacillus* sp. L81[i,j,m]<br><br>*Aeromonas* sp. AMG272[a,d,h,j] | Biotic<br>*Xanthomonas campestris*<br><br>Abiotic<br>*Salinity* | $F_v/F_m$ | L81 biotic ↑ | García-Cristobal et al., 2015 |
| | | | | L81 abiotic = | |
| | | | | AMG272 biot. ↑ | |
| | | | | AMG272 abiot. = | |
| | | | $\Phi_{PSII}$ | L81 biotic = | |
| | | | | L81 abiotic = | |
| | | | | AMG272 biot. = | |
| | | | | AMG272 abiot. = | |
| | | | NFQ | L81 biotic = | |
| | | | | L81 abiotic = | |
| | | | | AMG272 biot. = | |
| | | | | AMG272 abiot. = | |
| | | | Plant height | L81 biotic ↑ | |
| | | | | L81 abiotic ↓ | |
| | | | | AMG272 biot. ↑ | |
| | | | | AMG272 abiot. = | |
| | | | Guaiacol peroxidase | L81 biotic = | |

| | | | | | |
|---|---|---|---|---|---|
| | | | | L81 abiotic ↑ | |
| | | | | AMG272 biot. = | |
| | | | | AMG272 abiot. ↑ | |
| | | | Glutathione reductase | L81 biotic ↓ | |
| | | | | L81 abiotic = | |
| | | | | AMG272 biot. ↓ | |
| | | | | AMG272 abiot. = | |
| | | | Ascorbate peroxidase | L81 biotic ↑ | |
| | | | | L81 abiotic ↑ | |
| | | | | AMG272 biot. ↑ | |
| | | | | AMG272 abiot. ↑ | |
| | | | Superoxide dismutase | L81 biotic ↓ | |
| | | | | L81 abiotic = | |
| | | | | AMG272 biot. = | |
| | | | | AMG272 abiot. ↑ | |
| *Oryza sativa* | *Bacillus* sp. JIZ13[a,h,k,m] | Abiotic<br>*Salinity* | Shoot/Root height | ↑ | Wang et al., 2023 |
| | | | Total dry weight | ↑ | |
| | | | Relative water content | ↑ | |
| | | | Total chlorophyll | ↑ | |
| | | | $CO_2$ assimilation rates | ↑ | |
| | | | Transpiration rates | ↑ | |
| | | | Stomatal conductance | ↑ | |
| | | | Proline | ↑ | |
| | | | Sugar | ↑ | |
| | | | Protein | ↓ | |
| | | | $H_2O_2$ concentration | ↓ | |
| | | | $O_2$- concentration | ↓ | |
| | | | Malondealdehyde | ↓ | |
| | | | Catalase | = | |
| | | | Superoxide dismutase | ↑ | |
| | | | Peroxidase | ↑ | |
| | | | Soil enzymes (invertase, protease, urease, catalase) | ↑ | |
| *Raphanus sativus* | *Azospirillum brasilense*[c,h,p,q] | Abiotic<br>Flooding | Stomatal conductance | ↑ | Salazar-Garcia et al., 2022 |
| | | | Chlorophyll index | ↑ | |
| | | | $F_v/F_m$ | ↑ | |
| | | | PQ | ↑ | |
| | | | NPQ | = | |
| | | | ETR | = | |
| | | | Tuberous root diameter | ↑ | |
| | | | No. leaves | ↑ | |
| | | | Leaf area | ↑ | |
| | | | Total dry weight | ↑ | |
| *Salicornia ramosissima* | Consortium of<br><br>*Thalassospira australica* SRT8[a]<br><br>*Pseudarthrobacter oxydans* SRT15[c,h,j]<br><br>*Vibrio neocaledonicus* SRT1[c,h,j,k,m] | Abiotic<br>*Heavy metals (As, Cd, Cu, Co, Ni, Zn) & salinity* | Relative growth rates | ↑ | Mesa-Marín et al., 2020 |
| | | | Stem branches increment | ↑ | |
| | | | Water content | ↑ | |
| | | | $CO_2$ assimilation rates | ↑ | |
| | | | Stomatal conductance | = | |
| | | | Intercellular $CO_2$ conc. | ↓ | |
| | | | Water use efficiency | ↑ | |
| | | | $F_m$ | ↑ | |
| | | | $F_v/F_m$ | ↑ | |
| | | | Absorbed energy flux | = | |
| | | | Trapped energy flux | = | |

| | | | | | |
|---|---|---|---|---|---|
| | | | ET energy flux** | = | |
| | | | Dissipated energy flux | ↓ | |
| | | | Ca in leaves | = | |
| | | | Ca in roots | = | |
| | | | K in leaves | = | |
| | | | K in roots | = | |
| | | | Mg in leaves | = | |
| | | | Mg in roots | = | |
| | | | Mn in leaves | = | |
| | | | Mn in roots | ↓ | |
| | | | Na in leaves | ↑ | |
| | | | Na in roots | = | |
| | | | P in leaves | = | |
| | | | P in roots | = | |
| | | | As in leaves | = | |
| | | | As in roots | = | |
| | | | Cd in leaves | = | |
| | | | Cd in roots | = | |
| | | | Cu in leaves | = | |
| | | | Cu in roots | = | |
| | | | Ni inleaves | = | |
| | | | Ni in roots | = | |
| | | | Pb in leaves | = | |
| | | | Pb in roots | = | |
| | | | Zn in leaves | = | |
| | | | Zn in roots | = | |
| *Sambucus williamsii* | *Acinetobacter calcoaceticus* X128[e,h,q] | Abiotic *Light Drought* (LD) *Moderates Drougth* (MD) *Severe Drought* (SD) | $CO_2$ assimilation rates | LD; MD; SD ↑ | Liu et al., 2019a |
| | | | Stomatal conductance | LD; MD; SD ↑ | |
| | | | Intercellular $CO_2$ concentration | LD ↑ | |
| | | | | MD; SD = | |
| | | | Relative water content | LD; MD; SD ↑ | |
| | | | Cytokinins in roots | LD; MD; SD = | |
| | | | Cytokinins in shoots | LD; MD; SD ↑ | |
| | | | ABA in roots | LD; MD ↑ | |
| | | | | SD = | |
| | | | ABA in shoots | LD; MD; SD ↑ | |
| | | | Relative conductivity | LD = | |
| | | | | MD; SD ↓ | |
| | | | Shoot dry weight | LD; MD; SD ↑ | |
| | | | Root dry weight | LD; MD ↑ | |
| | | | | SD = | |
| *Sambucus williamsii* | Acinetobacter calcoaceticus X128[e,h,q] | Abiotic *Drought & recovery* | $CO_2$ assimilation rates | day 30 & 36 ↑ | Liu et al., 2019b |
| | | | | during recovery = | |
| | | | Stomatal conductance | day 24, 30, 36 ↑ | |
| | | | | during recovery ↑ | |
| | | | Intercellular $CO_2$ concentration | day 6-24 ↑ | |
| | | | | day 36 ↓ | |
| | | | | during recovery ↓ | |
| | | | Total chlorophyll | day 6-36 = | |
| | | | | during recovery ↓ | |
| | | | | | |

| | | | | | | |
|---|---|---|---|---|---|---|
| *Secale cereale* | *Pseudomonas putida* UW4[h] | Abiotic<br>Petroleum Hydrocarbon | Shoot length | | = | Gurska et al., 2015 |
| | | | Root length | | = | |
| | | | Root architecture | | ↑ | |
| | | | NPQ | | ↓ | |
| | | | $F_v/F_m$ | | = | |
| | | | $F_{PSII}$ | | = | |
| *Solanum lycopersicum* recombinant inbred lines (RIL20, RIL40, RIL66, RIL100) and Baludo | *Variovorax paradoxus* 5C-2 | Abiotic<br>*Drought* | Shoot dry weight | B, RIL20, 40, 100 | = | Calvo-Polanco et al., 2016 |
| | | | | RIL66 | ↑ | |
| | | | $CO_2$ assimilation rates | All inbred lines | = | |
| | | | Total chlorophyll | All inbred lines | = | |
| | | | Oxidative damage to lipids | All inbred lines | = | |
| | | | Proline content | B, RIL20, 40, 100 | = | |
| | | | | RIL66 | ↓ | |
| | | | Root hydraulic conductivity | RIL40, 66, 100 | = | |
| | | | | B | ↓ | |
| | | | | RIL20 | ↑ | |
| | | | PIP1 proteins | All inbred lines | = | |
| | | | PIP2 proteins | B, RIL100 | = | |
| | | | | RIL66 | ↓ | |
| | | | | RIL20, 40 | ↑ | |
| | | | Phosphorilated PIP2 proteins | B, RIL20, 40, 100 | = | |
| | | | | RIL66 | ↓ | |
| *Solanum tuberosum* | *Bacillus pumilus* DH-11[a,h,k,l]<br><br>*Bacillus firmus* 40[a,h,k,l] | Abiotic<br>*Drought, Salinity, Heavy metals (Zn)* | $F_v/F_m$ | DH-11 | ↑ | Gururani et al., 2013 |
| | | | | 40 | ↑ | |
| | | | Performance index | DH-11 | ↑ | |
| | | | | 40 | ↑ | |
| | | | $H_2O_2$ | DH-11 | ↓ | |
| | | | | 40 | ↓ | |
| | | | Plant height, leaves & tubers N°, tuber/plant yield | DH-11 | ↑ | |
| | | | | 40 | ↑ | |
| | | | Ascorbate peroxide | DH-11 | ↑ | |
| | | | | 40 | ↑ | |
| | | | Catalase | DH-11 | ↑ | |
| | | | | 40 | ↑ | |
| | | | Superoxide dismutase | DH-11 | ↑ | |
| | | | | 40 | ↑ | |
| | | | Glutathione reductase | DH-11 | ↑ | |
| | | | | 40 | ↑ | |
| | | | Dehydroascorbate reductase | DH-11 | ↑ | |
| | | | | 40 | ↑ | |
| | | | Proline | DH-11 | ↑ | |
| | | | | 40 | ↑ | |
| *Trema micrantha*<br><br>*Cariniana estrellensis* | *Azospirillum brasilense* Ab-V5[j]<br><br>*Bacillus* sp. (BA)[h]<br><br>*Azomonas* sp. (AM)<br><br>*Azorhizophillus* sp. (AR) | Abiotic<br>*Drought* | Water potential | Ab-V5 in *T.m.* | = | Nunes Tiepo et al., 2018 |
| | | | | Ab-V5 in *C.e.* | = | |
| | | | | BA in *T.m.* | = | |
| | | | | BA in *C.e.* | = | |
| | | | | AM in T.m. | = | |
| | | | | AM in *C.e.* | = | |
| | | | | AR in *T.m.* | ↑ | |
| | | | | AR in *C.e.* | = | |
| | | | Proline | Ab-V5 in *T.m.* | ↓ | |

| | | | | | | |
|---|---|---|---|---|---|---|
| | | | | Ab-V5 in *C.e.* | = | |
| | | | | BA in *T.m.* | = | |
| | | | | BA in *C.e.* | = | |
| | | | | AM in T.m. | ↓ | |
| | | | | AM in *C.e.* | = | |
| | | | | AR in *T.m.* | ↓ | |
| | | | | AR in *C.e.* | = | |
| | | | Thiobarbituric acid reactive substances | Ab-V5 in *T.m.* | = | |
| | | | | Ab-V5 in *C.e.* | = | |
| | | | | BA in *T.m.* | ↓ | |
| | | | | BA in *C.e.* | = | |
| | | | | AM in T.m. | = | |
| | | | | AM in *C.e.* | = | |
| | | | | AR in *T.m.* | ↑ | |
| | | | | AR in *C.e.* | ↑ | |
| | | | $H_2O_2$ | Ab-V5 in *T.m.* | = | |
| | | | | Ab-V5 in *C.e.* | = | |
| | | | | BA in *T.m.* | ↓ | |
| | | | | BA in *C.e.* | = | |
| | | | | AM in T.m. | ↓ | |
| | | | | AM in *C.e.* | = | |
| | | | | AR in *T.m.* | = | |
| | | | | AR in *C.e.* | = | |
| | | | Root & shoot dry weight | Ab-V5 in *T.m.* | = | |
| | | | | Ab-V5 in *C.e.* | = | |
| | | | | BA in *T.m.* | = | |
| | | | | BA in *C.e.* | = | |
| | | | | AM in T.m. | = | |
| | | | | AM in *C.e.* | = | |
| | | | | AR in *T.m.* | = | |
| | | | | AR in *C.e.* | = | |
| | | | Root:shoot ratio | Ab-V5 in *T.m.* | ↓ | |
| | | | | Ab-V5 in *C.e.* | = | |
| | | | | BA in *T.m.* | = | |
| | | | | BA in *C.e.* | = | |
| | | | | AM in T.m. | = | |
| | | | | AM in *C.e.* | = | |
| | | | | AR in *T.m.* | = | |
| | | | | AR in *C.e.* | ↓ | |
| | | | $CO_2$ assimilation rates | Ab-V5 in *T.m.* | = | |
| | | | | Ab-V5 in *C.e.* | ↑ | |
| | | | | BA in *T.m.* | = | |
| | | | | BA in *C.e.* | = | |
| | | | | AM in T.m. | = | |
| | | | | AM in *C.e.* | = | |
| | | | | AR in *T.m.* | = | |
| | | | | AR in *C.e.* | = | |
| | | | Stomatal conductance | Ab-V5 in *T.m.* | = | |
| | | | | Ab-V5 in *C.e.* | = | |
| | | | | BA in *T.m.* | = | |
| | | | | BA in *C.e.* | = | |
| | | | | AM in T.m. | = | |
| | | | | AM in *C.e.* | = | |
| | | | | AR in *T.m.* | = | |

| | | | | | | |
|---|---|---|---|---|---|---|
| | | | | AR in *C.e.* | = | |
| | | | Intercellular $CO_2$ concentration | Ab-V5 in *T.m.* | = | |
| | | | | Ab-V5 in *C.e.* | = | |
| | | | | BA in *T.m.* | = | |
| | | | | BA in *C.e.* | = | |
| | | | | AM in T.m. | = | |
| | | | | AM in *C.e.* | = | |
| | | | | AR in *T.m.* | = | |
| | | | | AR in *C.e.* | ↑ | |
| | | | Carboxylation efficiency | Ab-V5 in *T.m.* | = | |
| | | | | Ab-V5 in *C.e.* | ↑ | |
| | | | | BA in *T.m.* | = | |
| | | | | BA in *C.e.* | = | |
| | | | | AM in T.m. | = | |
| | | | | AM in *C.e.* | = | |
| | | | | AR in *T.m.* | = | |
| | | | | AR in *C.e.* | = | |
| | | | Total soluble sugars in leaves | Ab-V5 in *T.m.* | = | |
| | | | | Ab-V5 in *C.e.* | = | |
| | | | | BA in *T.m.* | = | |
| | | | | BA in *C.e.* | = | |
| | | | | AM in T.m. | ↓ | |
| | | | | AM in *C.e.* | = | |
| | | | | AR in *T.m.* | = | |
| | | | | AR in *C.e.* | = | |
| | | | Total soluble sugars in roots | Ab-V5 in *T.m.* | ↓ | |
| | | | | Ab-V5 in *C.e.* | = | |
| | | | | BA in *T.m.* | ↓ | |
| | | | | BA in *C.e.* | = | |
| | | | | AM in T.m. | = | |
| | | | | AM in *C.e.* | = | |
| | | | | AR in *T.m.* | ↓ | |
| | | | | AR in *C.e.* | = | |
| | | | Starch in leaves | Ab-V5 in *T.m.* | = | |
| | | | | Ab-V5 in *C.e.* | ↑ | |
| | | | | BA in *T.m.* | = | |
| | | | | BA in *C.e.* | = | |
| | | | | AM in T.m. | = | |
| | | | | AM in *C.e.* | = | |
| | | | | AR in *T.m.* | = | |
| | | | | AR in *C.e.* | = | |
| | | | Starch in roots | Ab-V5 in *T.m.* | = | |
| | | | | Ab-V5 in *C.e.* | = | |
| | | | | BA in *T.m.* | = | |
| | | | | BA in *C.e.* | = | |
| | | | | AM in *T.m.* | ↑ | |
| | | | | AM in *C.e.* | ↑ | |
| | | | | AR in *T.m.* | = | |
| | | | | AR in *C.e.* | = | |
| | | | Protein in leaves | Ab-V5 in *T.m.* | = | |
| | | | | Ab-V5 in *C.e.* | ↓ | |
| | | | | BA in *T.m.* | = | |
| | | | | BA in *C.e.* | = | |
| | | | | AM in *T.m.* | = | |

| | | | | | |
|---|---|---|---|---|---|
| | | | AM in *C.e.* | = | |
| | | | AR in *T.m.* | = | |
| | | | AR in *C.e.* | ↓ | |
| *Trigonella foenum-graecum* | Consortium of [r]<br><br>*Azotobacter chroococcum*<br><br>*Enterobacter asburiae*<br><br>*Lactococcus lactis* | Abiotic<br>*Salinity (70, 150 mM)* | Shoot length | ↑ | Bisht et al., 2022 |
| | | | Root length | ↑ | |
| | | | Shoot dry weight | ↑ | |
| | | | Root dry weight | ↑ | |
| | | | Leaf area | ↑ | |
| | | | No. leaves | ↑ | |
| | | | $CO_2$ assimilation rates | ↑ | |
| | | | Stomatal conductance | ↑ | |
| | | | Transpiration rates | ↑ | |
| | | | Intercellular $CO_2$ concentration | ↑ | |
| | | | Carotenoids | ↑ | |
| | | | Chlorophyll a | ↑ | |
| | | | Chlorophyll b | = | |
| | | | Total chlorophyll | ↑ | |
| | | | N | ↑ | |
| | | | Protein | ↑ | |
| *Triticum aestivum* | Consortium of<br><br>*Bacillus* sp.[e,h,p,q]<br><br>*Azospirillum lipoferum*[e,h,p,q]<br><br>*Azospirillum brasilense*[e,h,p,q] | Abiotic<br>*Drought* | $CO_2$ assimilation rates | ↑ | Akhtar et al., 2021 |
| | | | Transpiration rates | ↑ | |
| | | | Stomatal conductance | ↑ | |
| | | | Proline | ↑ | |
| | | | Sugar | = | |
| | | | Protein | ↑ | |
| | | | Relative water content | ↑ | |
| | | | Chlorophyll a | ↑ | |
| | | | Chlorophyll b | ↑ | |
| | | | Carotenoids | ↑ | |
| | | | Peroxidase | ↑ | |
| | | | Catalase | ↑ | |
| | | | Superoxide dismutase | = | |
| | | | N in root | = | |
| | | | P inroot | ↑ | |
| | | | K in root | ↑ | |
| | | | N in shoot | = | |
| | | | P in shoot | ↑ | |
| | | | K in shoot | ↑ | |
| | | | N in grain | ↑ | |
| | | | P in grain | ↑ | |
| | | | K in grain | ↑ | |
| | | | Total dry weight | = | |
| | | | Yield | = | |
| | | | Indole acetic acid in grains | ↑ | |
| | | | Cytokinins in grains | ↑ | |
| | | | ABA in grains | ↑ | |
| | | | Electrolyte leakage | ↓ | |
| *Vitis vinifera* | *Bacillus phytofirmans* PsJN[a,h] | Abiotic<br>*Cold* | $CO_2$ assimilation rates | ↑ | Fernandez et al., 2012 |
| | | | Stomatal conductance | = | |
| | | | Intercellular $CO_2$ concentration | = | |
| | | | $\Phi_{PSII}$ | = | |
| | | | Total chlorophyll | = | |
| | | | Carotenoids | = | |

| | | | | | |
|---|---|---|---|---|---|
| | | | Starch | ↑ | |
| | | | Total soluble sugars | = | |
| | | | Pyruvate | = | |
| | | | Phosphoenolpyruvate | = | |
| | | | Relative concentration os sugars and sugar alcohols (non-bacterized vs. bacterized) | galactinol raffinose mannose maltose ↑ | |
| | | | Relative concentration phosphated and nucleotined intermediates (non-bacterized vs. bacterized) | mannose-6 -phosphate ↓ | |
| | | | | others = | |
| *Vitis vinifera* | *Bacillus phytofirmans* PsJN[a,h] | Abiotic *Cold* | $CO_2$ assimilation rates | ↑ | Ait Barka et al., 2006 |
| | | | Oxygen production | ↑ | |
| | | | Total dry weight | ↑ | |
| | | | Shoot dry weight | = | |
| | | | Root dry weight | ↑ | |
| | | | Electrolytes leakage | - | |
| | | | Proline in shoots | ↑ | |
| | | | Proline in leaves | ↑ | |
| | | | Proline in roots | ↑ | |
| | | | Starch in shoots | ↑ | |
| | | | Starch in leaves | ↑ | |
| | | | Starch in roots | = | |
| | | | Phenols | ↑ | |
| *Zea mays* | *Pseudomonas fluorescens* Aur6[h,m] | Abiotic *Metal working fluids* | $F_0$ | = | Grijalbo et al., 2013 |
| | | | $F_v/F_m$ | = | |
| | | | Hill reaction | ↑ | |
| | | | Chlorophyll a/b | ↓ | |
| | | | Total Chlorophyll | ↑ | |
| *Zea mays* cv Marzuka<br><br>*Zea mays* cv Kaleo | *Bacillus phytofirmans* PsJN[a,h]<br><br>*Enterobacter* sp. FD17[a,b,c,k] | Abiotic *Drought* | $CO_2$ assimilation rates | PsJN both cv ↑ | Naveed et al., 2014 |
| | | | | FD17 both cv ↑ | |
| | | | Stomatal conductance | PsJN both cv ↑ | |
| | | | | FD17 both cv = | |
| | | | Transpiration rates | PsJN both cv ↑ | |
| | | | | FD17 both cv ↑ | |
| | | | Vapour pressure deficit | PsJN both cv ↑ | |
| | | | | FD17 both cv = | |
| | | | Relative water content | PsJN both cv ↑ | |
| | | | | FD17 both cv ↑ | |
| | | | $F_v/F_m$ | PsJN both cv ↑ | |
| | | | | FD17 both cv ↑ | |
| | | | Membrane permeability | PsJN both cv ↓ | |
| | | | | FD17 both cv ↓ | |
| | | | Total chlorophyll | PsJN both cv ↑ | |
| | | | | FD17 both cv ↑ | |
| | | | No. Leaves | PsJN both cv ↑ | |
| | | | | FD17 cv M ↑ | |
| | | | | FD17 cv K = | |
| | | | Leaf area | PsJN both cv ↑ | |
| | | | | FD17 both cv ↑ | |
| | | | Shoot dry weight | PsJN both cv ↑ | |
| | | | | FD17 both cv ↑ | |
| | | | Root dry weight | PsJN both cv ↑ | |
| | | | | FD17 both cv ↑ | |

| | | | | | |
|---|---|---|---|---|---|
| *Zea mays* | *Bacillus megaterium*[s] | Abiotic<br>*Drought; High temperature* | Electrolyte leakage | ↓ | Romero-Munar and Aroca, 2023 |
| | | | Shoot dry weight | = | |
| | | | Root dry weight | = | |
| | | | Shoot water content | ↑ | |
| | | | Stomatal conductance | = | |
| | | | $\Phi_{PSII}$ | ↑ | |
| | | | $CO_2$ assimilation rates | = | |
| | | | Water use efficiency | ↓ | |
| | | | Osmotic hydraulic conductivity | ↑ | |
| | | | Hydrostatic root hydraulic conductivity | = | |
| | | | Sap ABA | = | |
| | | | Sab jasmonic acid | ↓ | |
| | | | Sap IAA | ↓ | |
| | | | Sap salycilic acid | ↓ | |
| | | | Sap Ja-Ile | = | |
| | | | Acquaporins | = | |
| *Zea mays cv FriedriXX*<br><br>*Zea mays cv FuturiXX* | *Azospirillum lipoferum* CRT1[e,h,p,q] | Abiotic<br>Flooding | $\Phi_{PSII}$ | ↑ in Friedri | Czarnes et al., 2020 |
| | | | | ↓ in Futuri | |
| | | | $CO_2$ assimilation rates | ↑ in Friedri | |
| | | | | ↓ in Futuri | |
| | | | Total chlorophyll | = | |
| | | | Root length | = | |
| | | | Mean root diameter | = | |
| | | | Surface area | = | |
| | | | Total dry weight | = | |

*only if Fe concentration is suboptimal
**Electron transport energy flux

[a]ACC deaminase
[b]Biocontrol
[c]Biofilm formation
[d]Chitinases production
[e]Cytokinins production
[f]Exopolysaccharide production
[g]Glucose/ABA sensing
[h]IAA production
[i]Induction of systemic resistance
[j]N fixation
[k]Phosphate solubilization
[l]Phytase production
[m]Siderophore production
[n]VOCs production
[o]Cd tolerance
[p]ABA production
[q]Osmolyte production
[r] Effect on root architechture
[s] Effect on root hydraulic properties
